# The Na^+^/K^+^ pump dominates control of glycolysis in hippocampal dentate granule cells

**DOI:** 10.7554/eLife.81645

**Published:** 2022-10-12

**Authors:** Dylan J Meyer, Carlos Manlio Díaz-García, Nidhi Nathwani, Mahia Rahman, Gary Yellen

**Affiliations:** 1 Department of Neurobiology, Harvard Medical School Boston United States; University of Vermont United States; The University of Texas at Austin United States

**Keywords:** glycolysis, Na-Ca exchange, Na-K pump, cytosolic NADH, neurons, Mouse

## Abstract

Cellular ATP that is consumed to perform energetically expensive tasks must be replenished by new ATP through the activation of metabolism. Neuronal stimulation, an energetically demanding process, transiently activates aerobic glycolysis, but the precise mechanism underlying this glycolysis activation has not been determined. We previously showed that neuronal glycolysis is correlated with Ca^2+^ influx, but is not activated by feedforward Ca^2+^ signaling (Díaz-García et al., 2021a). Since ATP-powered Na^+^ and Ca^2+^ pumping activities are increased following stimulation to restore ion gradients and are estimated to consume most neuronal ATP, we aimed to determine if they are coupled to neuronal glycolysis activation. By using two-photon imaging of fluorescent biosensors and dyes in dentate granule cell somas of acute mouse hippocampal slices, we observed that production of cytoplasmic NADH, a byproduct of glycolysis, is strongly coupled to changes in intracellular Na^+^, while intracellular Ca^2+^ could only increase NADH production if both forward Na^+^/Ca^2+^ exchange and Na^+^/K^+^ pump activity were intact. Additionally, antidromic stimulation-induced intracellular [Na^+^] increases were reduced >50% by blocking Ca^2+^ entry. These results indicate that neuronal glycolysis activation is predominantly a response to an increase in activity of the Na^+^/K^+^ pump, which is strongly potentiated by Na^+^ influx through the Na^+^/Ca^2+^ exchanger during extrusion of Ca^2+^ following stimulation.

## Introduction

Cellular energy consumption must be balanced by new energy production through the activation of metabolism. In neurons, energy consumption is strongest following excitation (Attwell and Iadecola, 2002; Attwell and Laughlin, 2001; Engl and Attwell, 2015; Howarth et al., 2012; Lennie, 2003; Yu et al., 2018). Accordingly, neuronal excitation triggers a transient activation of glycolysis, which is measured as an increase to neuronal cytoplasmic NADH:NAD^+^ (NADH_CYT_) that can last for several minutes after the excitatory event (Díaz-García et al., 2017; Díaz-García et al., 2021a). The specific mechanism that drives neuronal glycolysis activation has not been identified.

It seems highly plausible that glycolysis activation in neurons could be driven primarily by an increase in the activity of ion-ATPases, the ATP-powered ion transporters that move ions against their electrochemical gradients by (through a series of reaction steps; Post et al., 1965; Sen and Post, 1964) converting ATP into ADP and P_i_ (Biondo et al., 2021; Calì et al., 2018; Chemaly et al., 2018; Kaplan, 2002). Ion-ATPases are estimated to be responsible for most of the energy consumption associated with neuronal signaling and are closely coupled to glycolytic enzymes across many cell types (Ames, 2000; Dhar-Chowdhury et al., 2007; Dzeja and Terzic, 2003; Xu et al., 1995). Moreover, activation of glycolysis by increased ion-ATPase activity could be multimodal: a decrease to cellular ATP would stimulate glycolysis by mass action at the ATP-generating steps, while an increase to cellular ADP and P_i_ would stimulate glycolysis by allosterically activating phosphofructokinase and by mass action at both the glyceraldehyde-3-phosphate dehydrogenase/phosphoglycerate kinase enzyme complex and pyruvate kinase steps (Fothergill-Gilmore and Michels, 1993; Kemp and Foe, 1983; Nelson et al., 2008; Schöneberg et al., 2013; Tomokuni et al., 2010).

**Figure 1.**
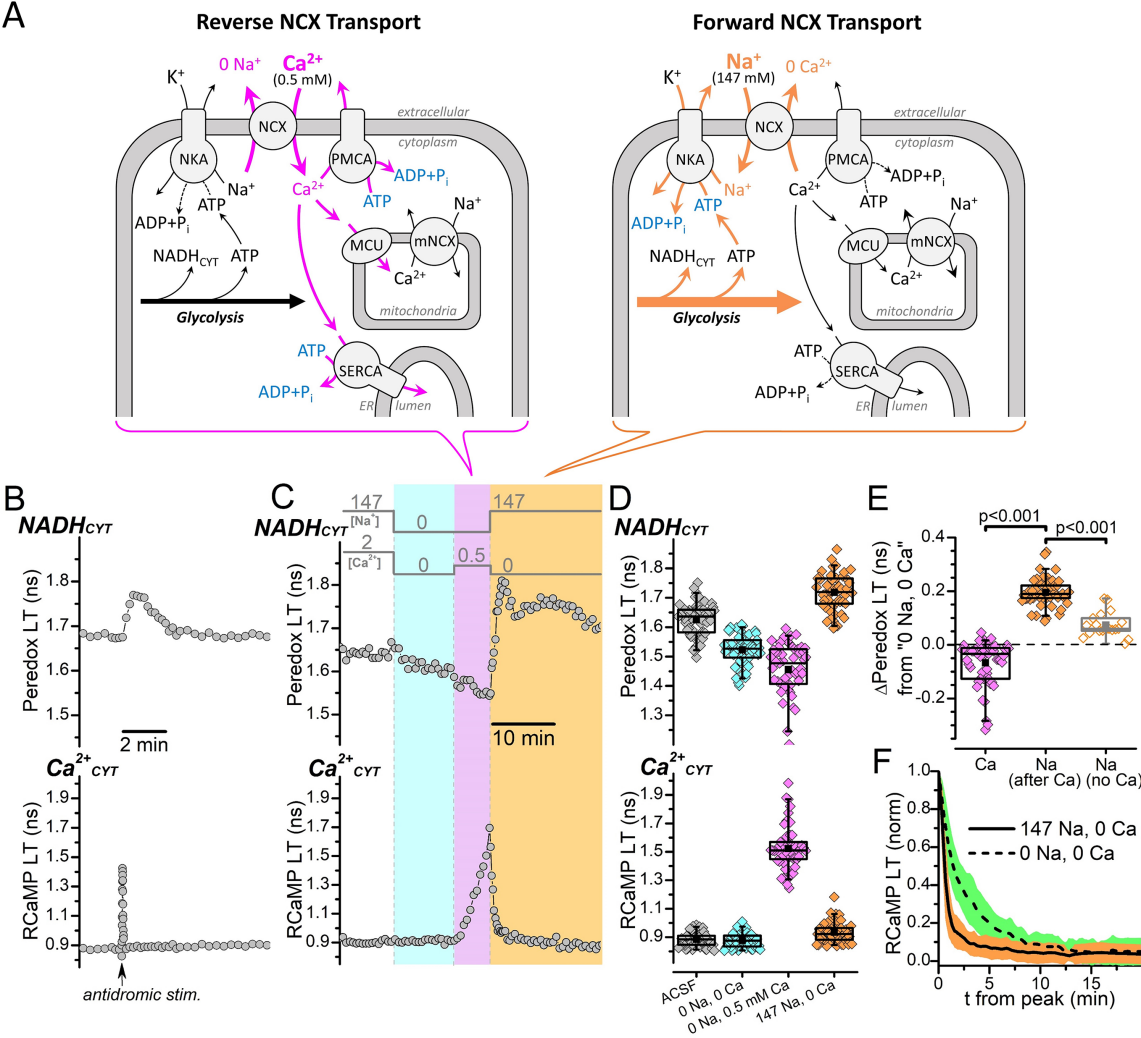
NADH_CYT_ production is strongly influenced by Na^+^, but not by Ca^2+^_CYT_. (**A**) Cartoon showing the NCX transport modes activated by different external [Na^+^] and [Ca^2+^] conditions and their expected effects on the activities of ion pumps and the production of NADH_CYT_ from glycolysis activation. Reverse NCX transport (*left schematic*) increases intracellular [Ca^2+^], which increases the activities Ca^2+^ pumps and Ca^2+^ transport into mitochondria (*magenta arrows*). Forward NCX transport (*right schematic*) increases intracellular [Na^+^], which increases the activity of the Na^+^/K^+^ pump (*orange arrows*). The bracket below each schematic indicates the NCX transport mode activated by the external solution changes in (**C**). Transport stoichiometries are not indicated. Abbreviations: Na^+^/Ca^2+^-exchanger (NCX), Na^+^/K^+^-ATPase (NKA), plasma membrane Ca^2+^-ATPase (PMCA), sarco-/endo-plasmic reticulum Ca^2+^-ATPase (SERCA), mitochondrial Ca^2+^ uniporter (MCU), mitochondrial Na^+^/Ca^2+^-exchanger (mNCX), endoplasmic reticulum (ER). (**B**) Representative fluorescence lifetime (LT) traces of Peredox (*top trace*) and RCaMP (*bottom trace*) from a DGC bathed in ACSF. Antidromic stimulation was delivered at the time point indicated by the arrow along the RCaMP trace, which transiently increases both NADH_CYT_ and Ca^2+^_CYT_. (**C**) Fluorescence LT traces of Peredox (*top*) and RCaMP (*bottom*) from a DGC showing how external Na^+^ and Ca^2+^ changes affect NADH_CYT_ and Ca^2+^_CYT_. The bars above the Peredox trace indicate the external [Na^+^] and [Ca^2+^]. NADH_CYT_ was decreased by switching the bath solution from ACSF (147 mM Na^+^ and 2 mM Ca^2+^) to a solution with nominally 0 Na^+^ and 0 Ca^2+^ (*cyan shading*). Ca^2+^_CYT_ was elevated by applying 0.5 mM Ca^2+^ with 0 Na^+^ to activate reverse NCX transport (*magenta shading*), and NADH_CYT_ decreased further. NADH_CYT_ was strongly increased after activating forward NCX transport by the subsequent removal of external Ca^2+^ and application of 147 mM Na^+^ (*orange shading*). (**D**) Box plots of the fluorescence LTs of Peredox (*top*) and RCaMP (*bottom*) showing the effects of the external Na^+^ and Ca^2+^ changes performed in panel C across many DGCs (n=53). The external bath conditions for each box plot are listed at the bottom of the RCaMP plot in chronological order from left to right. The colors of each box plot correspond to the colors indicated in (**C**). The mean Peredox LT values in each condition were: 1.63±0.06 ns in ACSF, 1.52±0.05 ns in 0 Na^+^ and 0 Ca^2+^, 1.46±0.09 ns in 0 Na and 0.5 mM Ca^2+^, and 1.72±0.06 ns in 147 mM Na^+^ and 0 Ca^2+^. The mean RCaMP LT values in each condition were: 0.88±0.05 ns in ACSF, 0.88±0.05 ns in 0 Na^+^ and 0 Ca^2+^, 1.52±0.15 ns in 0 Na^+^ and 0.5 mM Ca^2+^, and 0.93±0.07 ns in 147 mM Na^+^ and 0 Ca^2+^. (**E**) Changes to the Peredox LT relative to the 0 Na^+^ and 0 Ca^2+^ condition, after either a Ca^2+^_CYT_ elevation from reverse NCX transport (Ca, black box, magenta filled diamonds), an influx of Na^+^ due to forward NCX transport (Na after Ca, black box, orange filled diamonds), or application of Na^+^ without forward NCX (Na no Ca, gray box, orange open diamonds). The mean Peredox LT changes were: –0.07±0.08 ns (n=53) for Ca^2+^_CYT_ elevation, 0.20±0.05 ns (n=53) for Na^+^ influx via forward NCX, and 0.07364±0.04565 ns (n=19) for Na^+^ application without forward NCX. Statistical significance between ‘Ca’ and ‘Na after Ca’ is indicated by a paired Wilcoxon test and between ‘Na after Ca’ and ‘Na no Ca’ by a Mann-Whitney test. (**F**) Effect of external Na^+^ on the return of Ca^2+^_CYT_ to baseline following a reverse NCX transport-mediated Ca^2+^ influx. The mean decay of the RCaMP LT following the Ca^2+^_CYT_ increase (normalized to the peak RCaMP LT value) is shown when the external solution contained either 147 mM Na^+^ (*solid line, orange SD shading*, n=53) or 0 Na^+^ (*dashed line, green SD shading*, n=49). Decay data in 147 mM Na^+^ were from the same DGCs as in (**D**) and (**E**), while data in 0 Na^+^ were from the same DGCs as Figure 1—figure supplement 1B; a representative trace of this experiment is shown in Figure 1—figure supplement 1A.

There are several ion-ATPases (also called ion pumps) that regulate neuronal Na^+^ and Ca^2+^ changes (Figure 1A). Na^+^ is handled exclusively by the Na^+^/K^+^-ATPase (or Na^+^/K^+^ pump), which uses 1 ATP molecule to export 3 Na^+^ and import 2 K^+^ across the plasma membrane. Ca^2+^ is handled by a diverse set of active transporters, including the plasma membrane Ca^2+^-ATPase (PMCA), which exports 1 Ca^2+^ across the plasma membrane per ATP (Niggli et al., 1982; Thomas, 2009), and the sarco-/endo-plasmic reticulum Ca^2+^-ATPase (SERCA), which pumps 2 Ca^2+^ into the endoplasmic reticulum lumen per ATP (Tran et al., 2009). Aside from ATPases, Ca^2+^ is also regulated by the Na^+^/Ca^2+^-exchanger (NCX) (Lee et al., 2009), a secondary active transporter that uses the electrochemical energy stored in the plasma membrane Na^+^ gradient (built by the Na^+^/K^+^ pump) to actively export 1 Ca^2+^ by importing 3 Na^+^. Any increase to neuronal Na^+^ or Ca^2+^ will increase the activity of their respective ion transporters.

**Figure 1—figure supplement 1.**
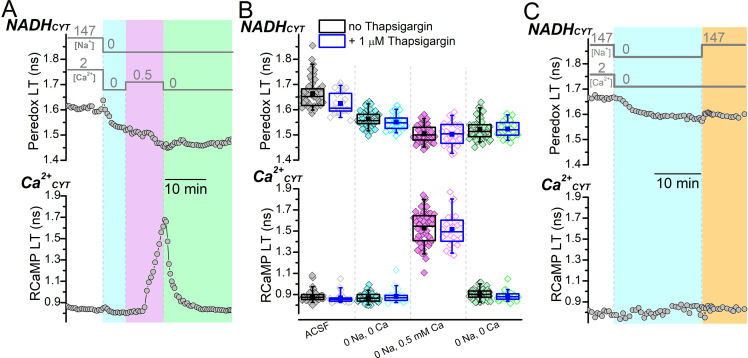
NADH_CYT_ production does not increase following external Ca^2+^ removal if external Na^+^ is absent. (**A**) Representative traces showing the effects of external [Na^+^] and [Ca^2+^] changes on Peredox (*top*) and RCaMP (*bottom*) lifetimes (LTs). The bars above the Peredox trace indicate the external [Na^+^] and [Ca^2+^]. After removing external Na^+^ and Ca^2+^ (*cyan shading*), Ca^2+^_CYT_ was increased by applying 0.5 mM Ca^2+^ in 0 Na^+^ to stimulate reverse NCX transport (*magenta shading*). Following the Ca^2+^_CYT_ increase, external Ca^2+^ was removed without re-addition of external Na^+^ (*green shading*) to follow the NADH_CYT_ and Ca^2+^_CYT_ levels without stimulating forward NCX transport. (**B**) Box plots of the fluorescence LTs of Peredox (*top*) and RCaMP (*bottom*) summarizing the effects of the external [Na^+^] and [Ca^2+^] changes performed in (**A**) across many DGCs. These experiments were performed either in the absence (*black boxes, filled symbols*, n=49) or presence of thapsigargin (*blue boxes, open symbols*, n=24). The external solution conditions for each box plot are listed at the bottom of the RCaMP plot in chronological order from left to right. The coloring of the data corresponds to the shading in (**A**). The mean Peredox and RCaMP LTs in each external condition are shown in Figure 1—figure supplement 1—source data 1. (**C**) Representative fluorescence LT traces of Peredox (*top*) and RCaMP (*bottom*) from a DGC showing the effect of external Na^+^ removal for 20 min (*cyan shading*) and re-addition (*orange shading*) in the absence of a Ca^2+^_CYT_ increase on NADH_CYT_. The external [Na^+^] and [Ca^2+^] are indicated by bars above the Peredox trace. Figure 1—figure supplement 1—source data 1.The mean Peredox and RCaMP lifetimes in each external condition for Figure 1—figure supplement 1B.

**Figure 1—figure supplement 2.**
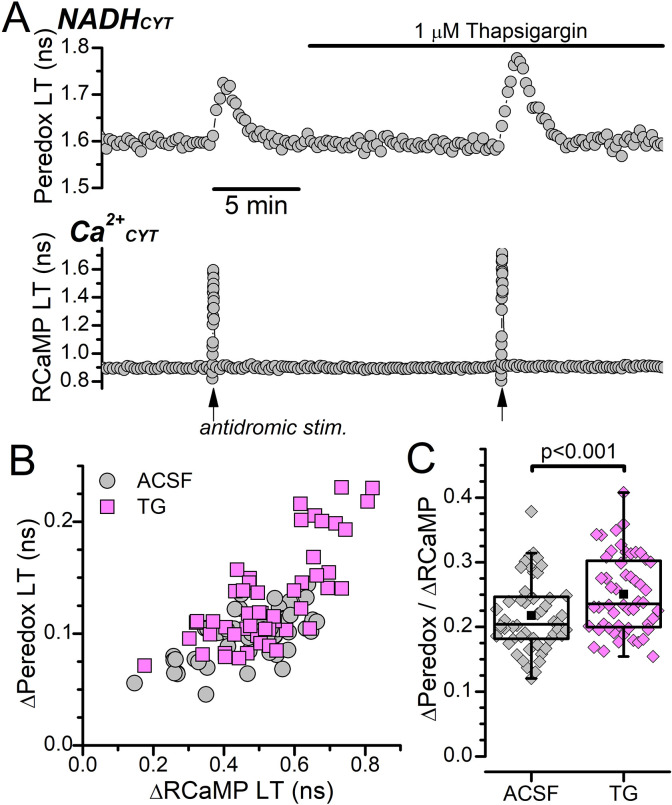
Effect of thapsigargin on antidromic stimulation-induced NADH_CYT_ and Ca^2+^_CYT_ transients. (**A**) Representative fluorescence lifetime (LT) traces of Peredox (*top*) and RCaMP (*bottom*) from a DGC bathed in ACSF showing transient increases to NADH_CYT_ and Ca^2+^_CYT_ induced by antidromic stimulation before and after application of 1 μM thapsigargin. Antidromic stimulation events are indicated by arrows below the RCaMP trace. (**B**) Scatterplot of the antidromic stimulation-induced transient changes in Peredox LT (∆Peredox) and RCaMP LT (∆RCaMP) before (ACSF, *gray circles*) or in the presence of 1 μM thapsigargin (TG, *magenta squares*) across many DGCs (n=52). (**C**) Box plots showing the effect of thapsigargin (TG) on the ratio of ∆Peredox to ∆RCaMP from the same DGCs as (**B**). The mean ∆Peredox/∆RCaMP was: 0.22±0.05 in ACSF and 0.25±0.06 with 1 μM TG. Statistical significance of a paired sample t-test is indicated.

**Figure 1—figure supplement 3.**
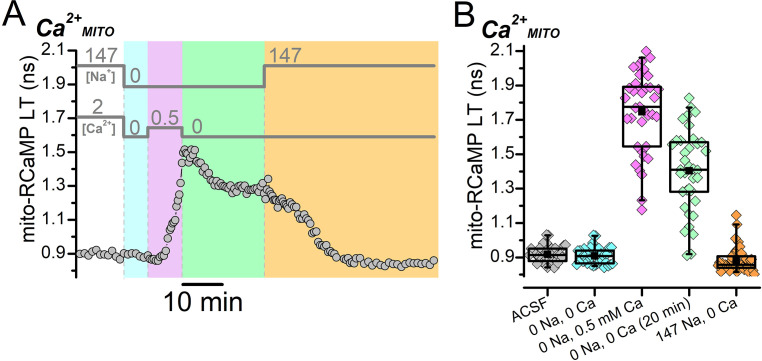
Ca^2+^ entry from reverse NCX transport increases Ca^2+^_MITO_, which depends on Na^+^ for removal. (**A**) Representative fluorescence lifetime (LT) trace of mito-RCaMP from a DGC showing how Ca^2+^_MITO_ is affected by changes to the external [Na^+^] or [Ca^2+^] (indicated by the bars above the trace). Following the removal of external Na^+^ and Ca^2+^ (*cyan shading*), addition of 0.5 mM Ca^2+^ in the absence of Na^+^ (*magenta shading*) to stimulate reverse NCX transport increased Ca^2+^_MITO._ The mito-RCaMP LT partially returned to baseline from the subsequent removal of external Ca^2+^ to turn off reverse NCX transport (*green shading*) but did not return completely to baseline until external Na^+^ was applied (orange shading). (**B**) Box plots showing the changes in mito-RCaMP LT induced by the external [Na^+^] or [Ca^2+^] changes performed in (**A**), across many DGCs (n=37). The external [Na^+^] and [Ca^2+^] conditions for each box plot are listed along the bottom in chronological order from left to right. The coloring of the data corresponds to the shading in (**A**). The LT values for the ‘0 Na, 0 Ca (20 min)’ condition (*green symbols*) were taken 20 min after removing 0.5 mM external Ca^2+^ in the absence of external Na^+^ (i.e., at the end of the *green shading* in **A**). The mean mito-RCaMP LT values in each condition are listed in Figure 1—figure supplement 3—source data 1. Figure 1—figure supplement 3—source data 1.The mean Peredox and RCaMP lifetimes in each external condition for Figure 1—figure supplement 3B.

Transient increases to neuronal cytoplasmic Ca^2+^ (Ca^2+^_CYT_) induced by excitation are positively correlated with transient increases in subsequent NADH_CYT_ production (Díaz-García et al., 2017; Díaz-García et al., 2021a); in other words, stronger stimulations evoke larger increases to both Ca^2+^_CYT_ and NADH_CYT_. At first glance, this positive correlation could be (mis)interpreted as glycolysis being driven primarily by an increase in the activities of Ca^2+^ pumps (PMCA and SERCA) as Ca^2+^ is pumped from the cytoplasm. However, this readout of neuronal NADH_CYT_ and Ca^2+^_CYT_ lacks information about stimulation-induced increases to intracellular Na^+^, which also likely covary with stimulation strength, so a contribution of increased Na^+^/K^+^ pump activity to glycolysis activation cannot be excluded.

The Na^+^/K^+^ pump is primed to be the predominant driving force underlying neuronal glycolysis activation. Many reports estimate that the Na^+^/K^+^ pump consumes ~50% of total brain energy (Ames, 2000; Astrup et al., 1981; Engl and Attwell, 2015; Milligan and McBride, 1985; Whittam, 1962) and uses substantially more ATP than Ca^2+^ pumps both during neurotransmission and at rest (Attwell and Laughlin, 2001; Harris et al., 2012; Rolfe and Brown, 1997). Furthermore, since Ca^2+^_CYT_ regulation in some neurons depends strongly on the NCX (Lee et al., 2009), a transient Ca^2+^_CYT_ increase could indirectly elevate intracellular [Na^+^], which would potentiate any increase in Na^+^/K^+^ pump activity due to channel-mediated Na^+^ entry.

To shed light on the mechanism of neuronal glycolysis activation, we investigated the coupling of Na^+^/K^+^ pump and Ca^2+^ pump activities to NADH_CYT_ production in the somas of hippocampal dentate granule cells (DGCs) within acute brain slices by using two-photon fluorescence imaging of genetically encoded fluorescent biosensors and fluorescent dyes. The data provide compelling evidence indicating that increased activity of the Na^+^/K^+^ pump is the predominant driver of neuronal glycolysis activation, whereas increased activities of Ca^2+^ pumps appear to be negligibly coupled to glycolysis.

## Results

### Cytoplasmic NADH production is strongly influenced by Na^+^, but not Ca^2+^

We simultaneously monitored changes in neuronal metabolism and ion fluxes in DGC somas by expressing Peredox and RCaMP, two genetically encoded fluorescent biosensors that report NADH:NAD^+^ (NADH_CYT_) and free [Ca^2+^], respectively (Akerboom et al., 2013; Hung et al., 2011; Mongeon et al., 2016). Analyte binding to Peredox (NADH in competition with NAD^+^) or RCaMP (Ca^2+^) alters each sensor’s fluorescence lifetime (LT), the average time that the fluorophore spends in the excited state prior to its return to the ground state (Lakowicz, 2006). The fluorescence LTs of Peredox and RCaMP are increased (i.e., extended in average duration) by NADH_CYT_ or Ca^2+^_CYT_ increases, respectively.

Our previous observations demonstrated that NADH_CYT_ increases are strongly tied to glycolysis (Díaz-García et al., 2017; Díaz-García et al., 2021a) and occur regardless of changes in mitochondrial metabolism: electrical-stimulation-induced increases to NADH_CYT_ were substantially diminished by iodoacetic acid (an inhibitor of GAPDH) but not by AOA (an inhibitor of MAS) and were not affected by impaired NADH production in mitochondria (i.e., in the presence of inhibitors of both the mitochondrial pyruvate carrier and lactate dehydrogenase; Figure 2D in Díaz-García et al., 2021a). Thus, acute NADH_CYT_ changes arise directly from changes in glycolytic flux and are not strongly influenced by changes in NADH consumption by the mitochondria.

We began our investigations on the coupling of the Na^+^/K^+^ pump or Ca^2+^ pumps to glycolysis activation by recapitulating our previous findings: that electrical stimulation of DGCs transiently increases their NADH_CYT_ production (Díaz-García et al., 2017; Díaz-García et al., 2021a). Representative LT traces of Peredox and RCaMP, when both sensors were expressed in the cytoplasm of a DGC within a hippocampal slice that was bathed in ACSF (Figure 1B), show that an antidromic stimulation event delivered from an electrode placed in the hippocampal hilus causes both a fast transient increase of the RCaMP LT, reflecting a transient increase to Ca^2+^_CYT_, and a slower, longer-lasting transient increase of the Peredox LT, reflecting transient overproduction of NADH_CYT_ by glycolysis. But since neuronal stimulation triggers influxes of both Na^+^ and Ca^2+^, the glycolysis activation could result from increases to both Na^+^/K^+^ pump and Ca^2+^ pump activities.

To differentiate how changes in the activities of the Na^+^/K^+^ pump or Ca^2+^ pumps affect glycolysis, we measured how NADH_CYT_ production is affected by separate elevations of either intracellular Na^+^ or Ca^2+^_CYT_ (Figure 1C). If glycolysis is preferentially coupled to the activity of either the Na^+^/K^+^ pump or Ca^2+^ pumps, then NADH_CYT_ production should be coupled to changes in the levels of the transported ion (Na^+^ or Ca^2+^). Simultaneous removal of both Na^+^ and Ca^2+^ from the bath solution (by external ion substitution) decreased the Peredox LT from its baseline in ACSF (Figure 1C), which indicates a decrease to NADH_CYT_; we can attribute this NADH_CYT_ decrease entirely to the removal of Na^+^, since removing only Ca^2+^ slightly increases NADH_CYT_ (cf. Figure 6—figure supplement 1b in Díaz-García et al., 2021a). This NADH_CYT_ decrease suggests that the rate of glycolysis can be slowed by depleting the intracellular [Na^+^], which would decrease the activity of the Na^+^/K^+^ pump. The RCaMP LT was not affected by the removal of Na^+^ and Ca^2+^ (Figure 1C), indicating that Ca^2+^_CYT_ was maintained at or below resting levels, near the floor of RCaMP’s dynamic range (Akerboom et al., 2013).

Following the removal of external Na^+^ and Ca^2+^, we added each ion back one at a time to separately increase either intracellular Na^+^ or Ca^2+^. We increased Ca^2+^ by applying 0.5 mM external Ca^2+^ in the nominal absence of external Na^+^ to facilitate Ca^2+^ entry via reverse NCX transport, and the expected strong increase in Ca^2+^_CYT_ was confirmed by an increase in the RCaMP LT (Figure 1C, during the magenta shaded interval). However, this increase to Ca^2+^_CYT_ was not associated with an increase to NADH_CYT_. In fact, the opposite occurred: Ca^2+^_CYT_ elevation decreased the Peredox LT even further from the 0 Na^+^ 0 Ca^2+^ bath condition (possibly due to the extrusion of Na^+^ during reverse NCX; see Discussion). At this point, with Ca^2+^_CYT_ elevated, we removed 0.5 mM external Ca^2+^ and re-applied external Na^+^ to facilitate strong Na^+^ influx via forward NCX transport (in exchange for Ca^2+^_CYT_), which promptly increased NADH_CYT_. Following the external Na^+^ application, Ca^2+^_CYT_ returned to baseline levels (Figure 1C, orange shaded interval).

The decrease to Peredox LT by removing external Na^+^ and the increase to Peredox LT from re-applying external Na^+^, across many DGCs (Figure 1D), show that NADH_CYT_ is strongly influenced by Na^+^. Relative to the Peredox LT in the 0 Na^+^ 0 Ca^2+^ bath condition, the Peredox LT was *decreased* when Ca^2+^_CYT_ was elevated but *increased* following external Na^+^ re-application (Figure 1E), which suggests that glycolysis activation is strongly tied to the activity of the Na^+^/K^+^ pump, but not strongly tied to the activity of Ca^2+^ pumps (nor to the increase in Ca^2+^_CYT_ per se, e.g., via a signaling mechanism).

Our ion substitution experiments also hinted that both glycolysis activation and Ca^2+^_CYT_ regulation in DGCs are dependent on forward NCX transport. A Ca^2+^_CYT_ increase (from reverse NCX transport) returned faster to baseline levels if external Na^+^ was present (i.e., if forward NCX was activated, Figure 1F; and compare Figure 1C with Figure 1—figure supplement 1A), and NADH_CYT_ did not increase following a Ca^2+^_CYT_ increase if external Ca^2+^ was removed without re-applying external Na^+^ (Figure 1—figure supplement 1A and B). NADH_CYT_ production increased when external Na^+^ was removed and re-applied without previously elevating Ca^2+^_CYT_ (i.e., without activating forward NCX, Figure 1—figure supplement 1C and D), but this NADH_CYT_ increase was weaker than when external Na^+^ was re-applied while Ca^2+^_CYT_ was elevated (i.e., when forward NCX was activated, Figure 1D and E).

The inability of a Ca^2+^_CYT_ elevation to measurably stimulate NADH_CYT_ production in the absence of external Na^+^ suggests that any increase in activity of PMCA and SERCA to pump Ca^2+^ from the cytoplasm does not substantially activate glycolysis, even during the prolonged Ca^2+^_CYT_ increases in our ion substitution experiments. But maybe inhibiting one of these Ca^2+^ pumps, to divert more Ca^2+^_CYT_ to be extruded by the non-inhibited pump, could boost the activity of the other and consequently amplify its associated effect on glycolysis. We tested this possibility by inhibiting SERCA, which compared to PMCA has more specific pharmacology and uses less ATP per Ca^2+^ transported. Thapsigargin (1 μM, a specific SERCA inhibitor) increased both the NADH_CYT_ and Ca^2+^_CYT_ transients evoked by antidromic electrical stimulations in ACSF (Figure 1—figure supplement 2A and B) as well as the ∆Peredox/∆RCaMP ratio (Figure 1—figure supplement 2C). These effects are consistent with SERCA inhibition and a diversion of Ca^2+^_CYT_ to other clearance pathways that are either more closely coupled to glycolysis or that transport fewer ions per ATP utilized. However, reverse-NCX-mediated Ca^2+^_CYT_ increases in the presence of 1 μM thapsigargin (and absence of external Na^+^) still failed to increase NADH_CYT_ (Figure 1—figure supplement 1B), meaning that any putative amplification of PMCA activity after SERCA inhibition was still not enough to drive measurable glycolysis activation.

We have previously shown that electrical stimulation increases DGC mitochondrial Ca^2+^ (Ca^2+^_MITO_) from Ca^2+^ entry through the mitochondrial Ca^2+^ uniporter (MCU) (Díaz-García et al., 2021a). Could Ca^2+^_CYT_’s failure to activate NADH_CYT_ production during our ion substitution experiments be due to a lack of activation of some unknown Ca^2+^_MITO_-dependent process? This seems unlikely, as bath-applied 0.5 mM external Ca^2+^ in the absence of external Na^+^ increased the fluorescence LT of mitochondrially targeted RCaMP (Figure 1—figure supplement 3A and B), indicating that Ca^2+^_MITO_ is increased by reverse-NCX-mediated Ca^2+^ entry. Ca^2+^_MITO_ slightly decreased when external Ca^2+^ was removed, but complete return of Ca^2+^_MITO_ to baseline levels was not achieved until external Na^+^ was applied, demonstrating that Ca^2+^_MITO_ regulation is dependent on Na^+^, like Ca^2+^_CYT_.

In summary, these ion substitution experiments show that neuronal NADH_CYT_ production is closely coupled to the flux of Na^+^, and that a Ca^2+^_CYT_ elevation is not sufficient to elevate NADH_CYT_ regardless of whether it is pumped by SERCA, pumped by PMCA, or taken up into mitochondria via MCU. They also show that NADH_CYT_ production is activated more strongly by conditions that favor forward NCX transport that exchanges Ca^2+^_CYT_ for Na^+^, and that regulation of both DGC Ca^2+^_CYT_ and Ca^2+^_MITO_ depends on Na^+^. These observations suggest that neuronal glycolysis is both strongly coupled to the activity of the Na^+^/K^+^ pump and sensitive to Na^+^ import via forward NCX transport, which would become activated following an excitatory event where Ca^2+^_CYT_ must be removed.

### Malate-aspartate shuttle activity does not mask a Ca^2+^-induced increase to NADH_CYT_

**Figure 2.**
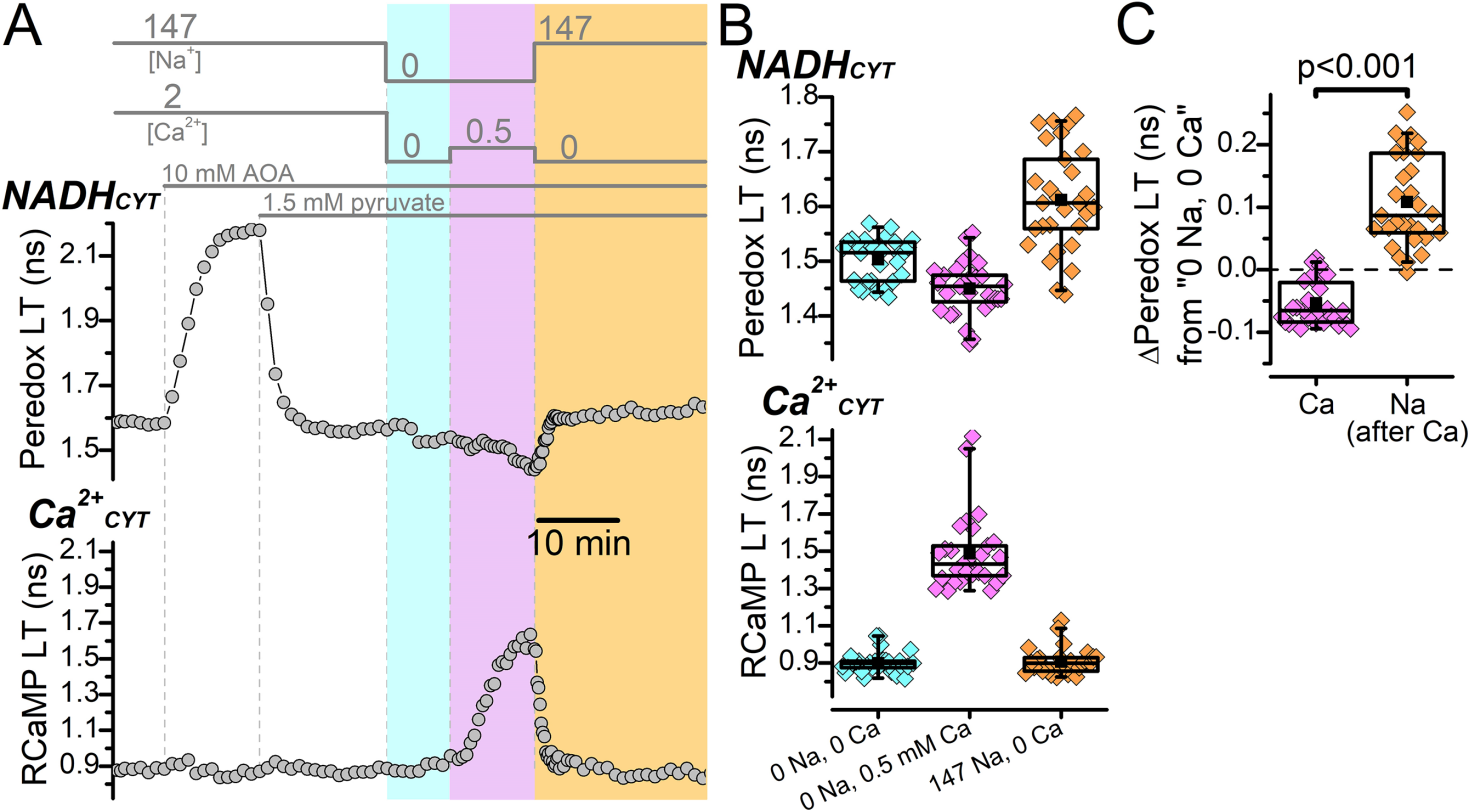
Malate-aspartate shuttle activity does not conceal a possible NADH_CYT_ increase produced by elevated Ca^2+^_CYT_. (**A**) Representative fluorescence lifetime (LT) traces of Peredox (*top*) and RCaMP (*bottom*) from a DGC. External solution changes are indicated by the bars above the Peredox trace. The recording begins in ACSF (147 mM Na^+^ and 2 mM Ca^2+^), where the addition of 10 mM AOA strongly increases the Peredox LT. Addition of 1.5 mM pyruvate reverted the Peredox LT approximately back to baseline. Following this, external Na^+^ and Ca^2+^ were removed (*cyan shading*), which slightly decreased the Peredox LT. The addition of 0.5 mM Ca^2+^ in 0 Na^+^ to stimulate reverse NCX transport increased the RCaMP LT and further decreased the Peredox LT (*magenta shading*). Subsequently, the removal of external Ca^2+^ and re-addition of external Na^+^ to stimulate forward NCX transport (*orange shading*) increased the Peredox LT and brought the RCaMP LT back to baseline. (**B**) Box plots of the Peredox (*top*) and RCaMP (*bottom*) fluorescence LTs in the different external solution conditions performed in (**A**), across many DGCs (n=29). The external [Na^+^] and [Ca^2+^] condition after applying AOA and pyruvate is listed across the bottom in chronological order from left to right. The coloring of the data corresponds to the shading in A. The mean Peredox and RCaMP LTs in each external condition were (in ns): 1.50±0.04 in 0 Na^+^ and 0 Ca^2+^, 1.45±0.05 in 0 Na^+^ and 0.5 mM Ca^2+^, and 1.61±0.09 in 147 Na^+^ and 0 Ca^2+^. The mean RCaMP LTs were (in ns): 0.90±0.06 in 0 Na^+^ and 0 Ca^2+^, 1.49±0.20 in 0 Na^+^ and 0.5 mM Ca^2+^, and 0.91±0.07 in 147 Na^+^ and 0 Ca^2+^. (**C**) Relative change to the Peredox fluorescence LT induced by the addition of 0.5 mM Ca^2+^ in the absence of external Na^+^ to stimulate reverse NCX transport (Ca) or 147 mM Na^+^ in the absence of external Ca^2+^ to stimulate forward NCX transport (Na). The data are from the same DGCs as in (**B**). The mean ∆Peredox values were: –0.05±0.04 ns for ‘Ca’ and 0.11±0.07 ns for ‘Na’. Statistical significance from a paired Wilcoxon test is indicated.

The malate-aspartate shuttle (MAS) is a major pathway that recycles NADH_CYT_ by transferring reducing equivalents from NADH_CYT_ to mitochondria (McKenna et al., 2006; Satrústegui and Bak, 2015). MAS activity is activated by Ca^2+^_CYT_ elevations but is reduced by Ca^2+^_MITO_ elevations (Contreras and Satrústegui, 2009; Satrústegui and Bak, 2015), which likely means that neuronal excitation would cause a brief MAS activation followed by a longer inhibition, mirroring the Ca^2+^_CYT_ and Ca^2+^_MITO_ transient time courses (Contreras and Satrústegui, 2009). But the state of MAS activity in our ion substitution experiments was uncertain since reverse NCX transport produced prolonged increases to both Ca^2+^_CYT_ and Ca^2+^_MITO_ (Figure 1 and Figure 1—figure supplement 3), so it remained a possibility that Ca^2+^_CYT_ activation of MAS could increase NADH_CYT_ recycling and conceal any small increase to NADH_CYT_ production due to increased activity of Ca^2+^ pumps. Therefore, we tested if a Ca^2+^_CYT_ elevation could increase NADH_CYT_ after MAS inhibition (Figure 2).

Representative fluorescence LT traces from a DGC show that Peredox LT was substantially increased by the application of AOA to block the shuttle’s aspartate aminotransferase enzyme (Figure 2A), as previously described, Díaz-García et al., 2017, which is consistent with an inhibition of NADH_CYT_ recycling and consequent elevation of the cytoplasmic NADH:NAD^+^. This strong increase to Peredox LT, which approaches the upper end of its dynamic range, precludes the ability to measure further NADH_CYT_ increases, so we applied exogenous pyruvate (1.5 mM) to promote the oxidation of NADH to NAD^+^ via lactate dehydrogenase and restore the Peredox LT approximately back to its baseline LT in ACSF. We then removed external Na^+^ and Ca^2+^, which decreased the Peredox LT from the AOA and pyruvate condition (Figure 2A); and then increased Ca^2+^_CYT_ by applying 0.5 mM external Ca^2+^ in zero external Na^+^ to drive reverse NCX transport. We confirmed the Ca^2+^_CYT_ increase from the increase to RCaMP LT, but the Ca^2+^_CYT_ elevation still did not coincide with an increase to NADH_CYT_. Rather, NADH_CYT_ decreased relative to the 0 Na^+^ 0 Ca^2+^ baseline (Figure 2B and C). This demonstrates that an increase to Ca^2+^_CYT_ still fails to observably stimulate NADH_CYT_ production even when NADH_CYT_ recycling by the MAS is attenuated. Following the Ca^2+^_CYT_ increase, we removed 0.5 mM external Ca^2+^ and re-applied external Na^+^ to activate forward NCX transport (Figure 2A), which increased the Peredox LT (Figure 2B and C). This shows again that NADH_CYT_ production is coupled to the flux of Na^+^.

Overall, these experiments argue against a concealment of Ca^2+^_CYT_-induced NADH_CYT_ production by a Ca^2+^_CYT_ activation of NADH_CYT_ recycling via the MAS and indicates, once again, that an increase to Na^+^/K^+^ pump activity is the major driver of glycolysis.

### Activation of glycolysis by Ca^2+^ is mediated by coupling between the Na^+^/Ca^2+^-exchanger and the Na^+^/K^+^ pump

The increase to NADH_CYT_ production by an influx of Na^+^ (Figure 1 and Figure 2) suggests that glycolysis activation in DGCs is strongly tied to an increase in the activity of the Na^+^/K^+^ pump. Also, the NADH_CYT_ increase induced by external Na^+^ application was stronger if Ca^2+^_CYT_ was elevated compared to when Ca^2+^_CYT_ was *not* elevated (Figure 1E), meaning that forward NCX transport, which trades Ca^2+^_CYT_ for Na^+^, can potentiate glycolysis activation. By considering these observations together with the apparent insensitivity of glycolysis to increases in Ca^2+^ pump activity, it seemed logical that a transient Ca^2+^_CYT_ elevation would activate glycolysis predominantly by stimulating Na^+^ influx through forward NCX transport, which would then increase the activity of the Na^+^/K^+^ pump.

**Figure 3.**
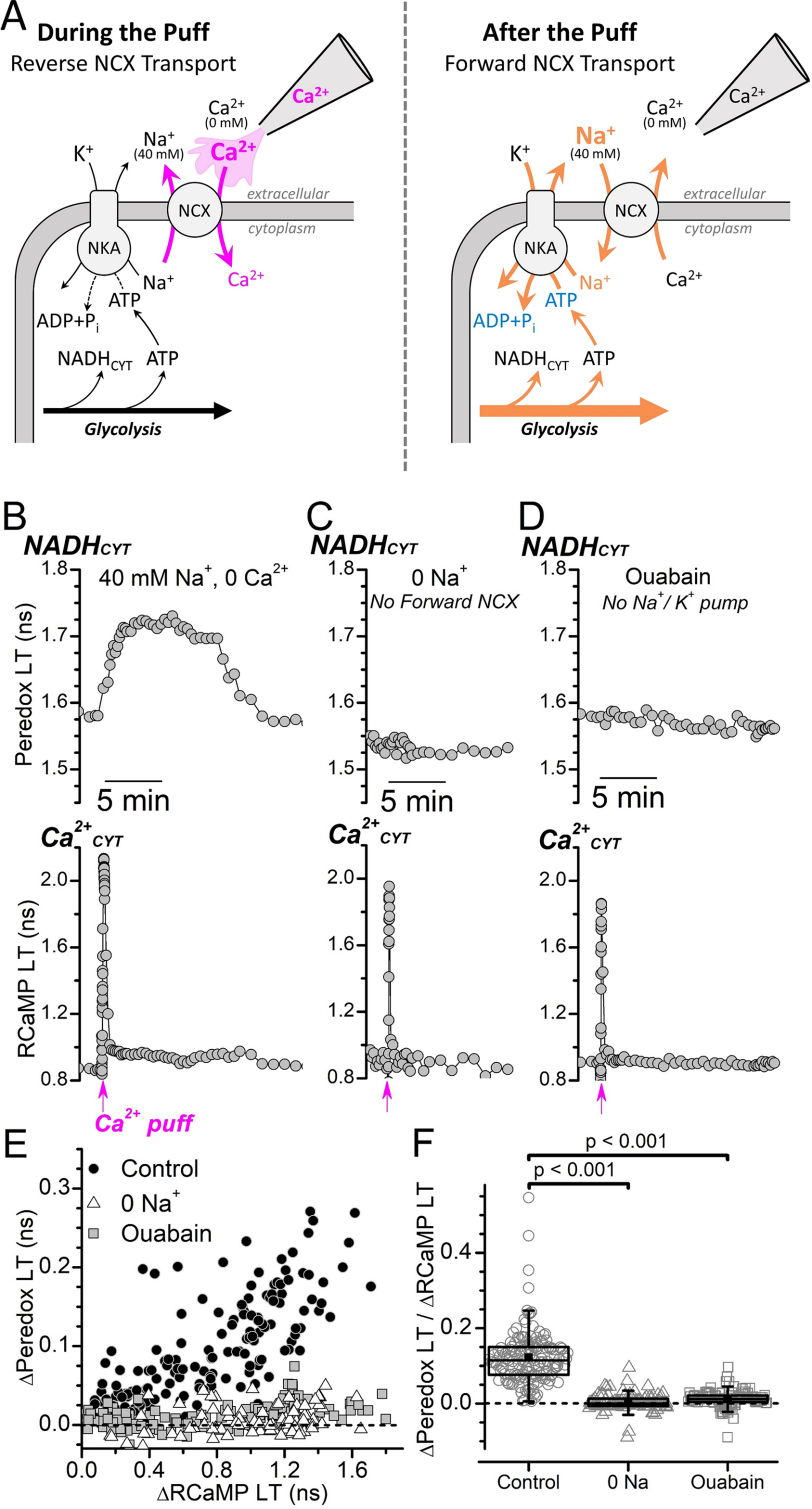
Ca^2+^_CYT_ transients induced by external Ca^2+^ puffs only increase NADH_CYT_ production when both forward NCX transport and Na^+^/K^+^ pump activity are intact. (**A**) Cartoon depicting the expected effect of a local external Ca^2+^ puff on ion transport by the NCX and subsequent activation of the Na^+^/K^+^ pump. (*Left*) A pipette containing Ca^2+^ is placed near the soma of a DGC within a slice that is bathed in solution containing 40 mM Na^+^ and 0 Ca^2+^ solution. A brief pulse of positive pressure is applied to the pipette to transiently increase the local external [Ca^2+^] and stimulate reverse NCX transport (*magenta arrows*), leading to Ca^2+^ import and an increase to Ca^2+^_CYT_. (*Right*) In the aftermath of the puff, the local external [Ca^2+^] decreases as the small volume of puffed Ca^2+^ mixes with large volume of the 40 mM Na^+^ 0 Ca^2+^ bath solution, which leads to forward NCX transport that stimulates Na^+^ extrusion and an increase to Na^+^/K^+^ pump activity (*orange arrows*). Abbreviations: Na^+^/Ca^2+^-exchanger (NCX), Na^+^/K^+^-ATPase (NKA). (**B–D**) Representative fluorescence lifetime (LT) traces of Peredox (*top traces*) and RCaMP (*bottom traces*) from a DGC bathed in either 40 mM Na^+^ and 0 Ca^2+^ solution (**B**, *Control*), 0 Na^+^ and 0 Ca^2+^ to block forward NCX transport (**C**, *0 Na^+^*), or in 40 mM Na^+^ and 0 Ca^2+^ with 5 mM ouabain to block the Na^+^/K^+^ pump (**D**, *Ouabain*). Puffs of Ca^2+^ were delivered at the timepoint indicated along the bottom of the RCaMP LT traces (*magenta arrows*). (**E**) Scatterplot of the Ca^2+^-puff-induced transient changes to the Peredox and RCaMP LTs of DGCs in 40 mM Na^+^ and 0 Ca^2+^ (*Control, black circles,* n=172), in 0 Na^+^ and 0 Ca^2+^ (*0 Na^+^, white triangles*, n=102), or in 40 mM Na^+^ and 0 Ca^2+^ with 5 mM ouabain (*Ouabain, gray squares*, n=114). The dashed line indicates where ∆Peredox=0. Analysis was restricted to recordings with transient RCaMP LT half-decay times <35 s. (**F**) Ratio of ∆Peredox to ∆RCaMP for the same Ca^2+^-puff-induced LT transients as in panel E, except excluding those recordings where the ∆RCaMP LT was <0.2 ns to avoid noisy ratio calculations due to small ∆RCaMP values. The dashed line indicates where ∆Peredox/∆RCaMP=0. The mean ∆Peredox/∆RCaMP in each condition was: 0.12±0.07 in 40 mM Na^+^ and 0 Ca^2+^ (*Control*, n=147), 0.01±0.02 in 0 Na^+^ and 0 Ca^2+^ (*0 Na^+^*, n=96), and 0.00±0.02 in 40 mM Na^+^ and 0 Ca^2+^ with 5 mM ouabain (*Ouabain*, n=99). Statistical significance from Mann-Whitney tests is indicated.

We sought for a way to directly test how NADH_CYT_ is affected by inhibition of the Na^+^/K^+^ pump or inhibition of forward NCX transport (Figure 3). Na^+^/K^+^ pumps are specifically inhibited by ouabain, although the rodent Na^+^/K^+^ pump α1-isozymes have relatively low sensitivity and require millimolar inhibitor concentrations for complete inhibition under physiological conditions (Blanco and Mercer, 1998; Marks and Seeds, 1978). Application of 2 mM ouabain to DGCs bathed in ACSF quickly led to Ca^2+^ overload and cell rupture (Figure 3—figure supplement 1), which made it impossible to measure how electrical stimulation-induced NADH_CYT_ transients are affected by complete Na^+^/K^+^ pump inhibition. Clearly, the activity of the Na^+^/K^+^ pump must be substantial, even at rest, but achieving complete Na^+^/K^+^ pump inhibition under physiological neuronal conditions is challenging. DGCs bathed in ACSF were more tolerant to application of low-dose (5 μM) ouabain, which inhibits only the rodent Na^+^/K^+^ pump α2- and α3-isozymes that have higher inhibitor sensitivities (Blanco and Mercer, 1998), but the stimulation-induced NADH_CYT_ transient was not eliminated (Figure 3—figure supplement 2). This result argues that in these cells, the α1 subunit is adequate to support the activation of glycolysis.

**Figure 3—figure supplement 1.**
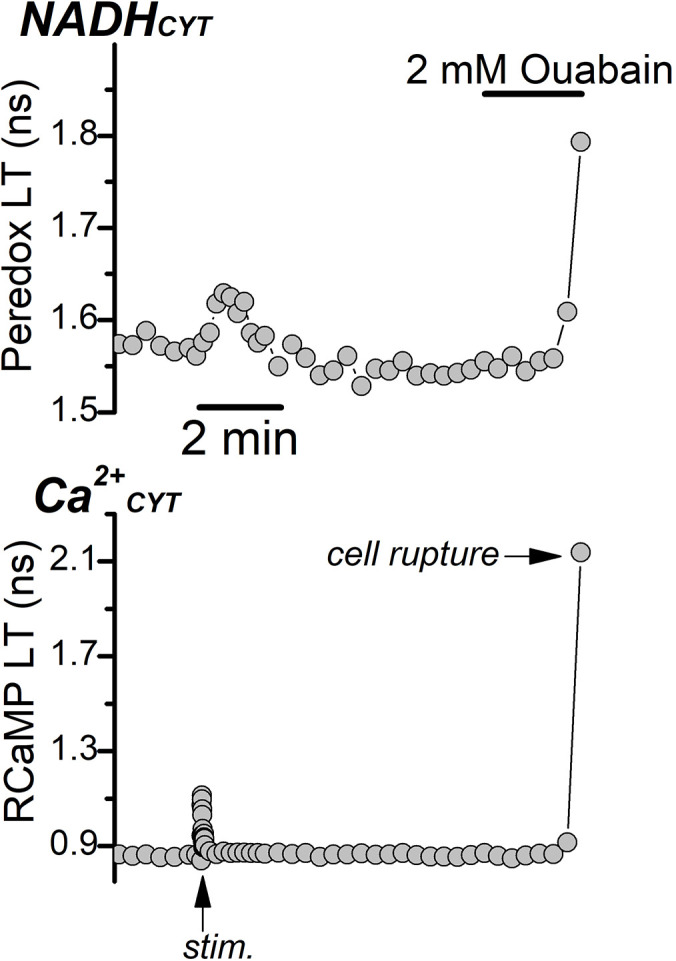
Complete Na^+^/K^+^ pump inhibition in ACSF leads to cell rupture. Representative fluorescence lifetime traces of Peredox (*top*) and RCaMP (*bottom*) from a DGC bathed in ACSF. Synaptic stimulation of the afferent fibers in the absence of synaptic blockers (*stim*, indicated by the arrow at the bottom of the RCaMP trace) transiently increased Ca^2+^_CYT_ and NADH_CYT_. Application of 2 mM ouabain irreversibly increased Ca^2+^_CYT_ and ruptured the DGCs (*cell rupture,* arrow in the RCaMP trace).

**Figure 3—figure supplement 2.**
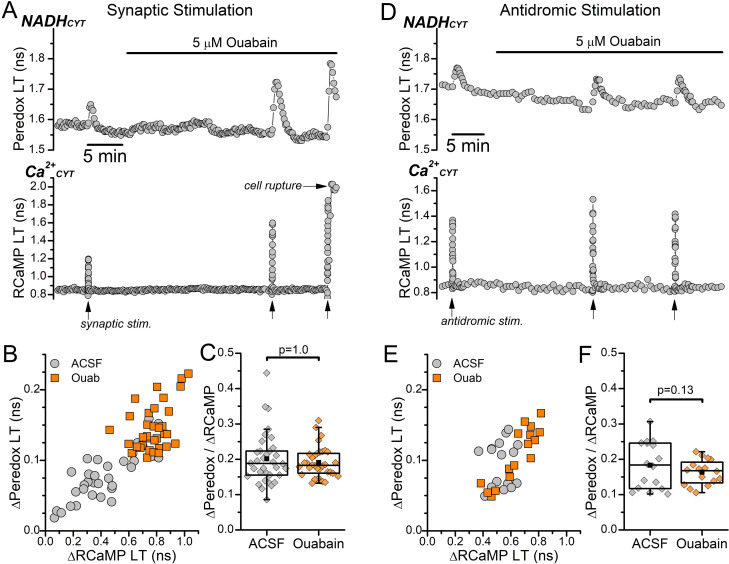
Effect of low dose (5 μM) ouabain on transient Peredox and RCaMP lifetime (LT) increases evoked by synaptic or antidromic stimulation. (**A–C**) Effect of low dose ouabain on synaptic stimulation-induced transients in the absence of synaptic blockers. (**A**) Representative fluorescence LT traces of Peredox (*top*) and RCaMP (*bottom*) from a DGC bathed in ACSF. The DGC was stimulated synaptically with an electrode placed in the afferent fibers at the timepoints indicated by the arrows along the bottom of the RCaMP LT trace. Application of 5 μM ouabain is indicated by the bar above the Peredox trace. Repetitive synaptic stimulations in ouabain often ruptured the DGCs (*cell rupture,* arrow in the RCaMP trace). (**B**) Scatterplot of the transient Peredox and RCaMP LT increases (n=34) induced by synaptic stimulation in ACSF (*ACSF, gray circles*) and in 5 μM ouabain (*Ouab, orange squares*). (**C**) The ∆Peredox/∆RCaMP ratio from synaptic stimulations in ACSF and with 5 μM ouabain, calculated from the same DGCs as in (**B**). The mean ∆Peredox/∆RCaMP in each condition was: 0.20±0.07 in ACSF and 0.19±0.04 in ouabain. Statistical significance from a paired Wilcoxon test is indicated. (**D–F**) Effect of low dose ouabain on antidromic stimulation-induced transients. (**D**) Representative fluorescence LT traces of Peredox (*top*) and RCaMP (*bottom*) from a DGC bathed in ACSF with synaptic blockers. The DGC was stimulated antidromically at the timepoints indicated by the arrows along the bottom of the RCaMP trace. Application of 5 μM ouabain is indicated by the bar above the Peredox trace. (**E**) Scatterplot of the transient Peredox and RCaMP LT increases (n=16) induced by antidromic stimulation in ACSF with synaptic blockers (*ACSF, gray circles*) and with 5 μM ouabain (*Ouab, orange squares*). (**F**) The ∆Peredox/∆RCaMP ratio from antidromic stimulations in ACSF and in 5 μM ouabain, calculated from the same DGCs as in (**E**). The mean ∆Peredox/∆RCaMP in each condition was: 0.18±0.07 in ACSF with synaptic blockers and 0.16±0.04 in ouabain. Statistical significance from a paired sample t-test is indicated.

We successfully established a condition amenable to millimolar ouabain application by reducing the external [Na^+^] to 40 mM and removing external Ca^2+^ to prevent unintended reverse NCX transport. Stimulation-induced Ca^2+^_CYT_ transients using an electrode were not possible for DGCs bathed in this condition, but we evoked similar Ca^2+^_CYT_ transients by applying puffs of extracellular Ca^2+^ to transiently produce reverse-NCX transport (Figure 3A). A brief puff of Ca^2+^ to a DGC within a slice bathed in 40 mM Na^+^ and 0 Ca^2+^ (Figure 3B) induced a fast transient increase to the RCaMP LT, reflecting a fast increase to Ca^2+^_CYT_, followed by a transient increase to the Peredox LT, reflecting an increase to NADH_CYT_. This shows that a transient Ca^2+^_CYT_ elevation can, indeed, activate glycolysis under these ionic conditions. But for a DGC within a slice that was instead bathed in zero external Na^+^ to prevent the NCX-mediated influx of Na^+^ in exchange for the elevated Ca^2+^_CYT_, a Ca^2+^ puff still evoked a fast Ca^2+^_CYT_ transient but did not increase NADH_CYT_ (Figure 3C). Similarly, the NADH_CYT_ increase that would normally follow the Ca^2+^ puff was prevented for a DGC within a slice bathed with 5 mM ouabain to block Na^+^/K^+^ pumps (Figure 3D).

A scatter plot of the transient Peredox LT and RCaMP LT changes that were evoked by Ca^2+^ puffs (Figure 3E) illustrates the positive correlation between NADH_CYT_ production and Ca^2+^_CYT_ increases for DGCs bathed in 40 mM Na^+^ and 0 Ca^2+^, where both forward NCX and Na^+^/K^+^ pump activities are intact. But NADH_CYT_ production was deeply attenuated when DGCs were bathed without external Na^+^ or with 5 mM ouabain, even with large elevations of Ca^2+^_CYT_ (∆RCaMP>1.2 ns). The mean ∆Peredox/∆RCaMP ratio (Figure 3F) for Ca^2+^-puff-evoked transients in 40 mM external Na^+^ (0.122±0.005, n=147) was reduced to nearly 0 after blocking forward NCX transport by removing external Na^+^ (0.003±0.002, n=96) or after blocking the Na^+^/K^+^ pump with ouabain (0.013±0.002, n=99).

These data demonstrate that Ca^2+^_CYT_ can activate glycolysis in the DGC soma if it is traded for Na^+^ through forward NCX transport, which increases the activity of the Na^+^/K^+^ pump. The lack of Ca^2+^-puff-induced NADH_CYT_ responses when either forward NCX transport or the Na^+^/K^+^ pump was blocked further substantiates that any increase in the activity of Ca^2+^ pumps has a negligible effect on glycolysis activation.

### Antidromic stimulation-induced Na^+^ transients depend on Ca^2+^

**Figure 4.**
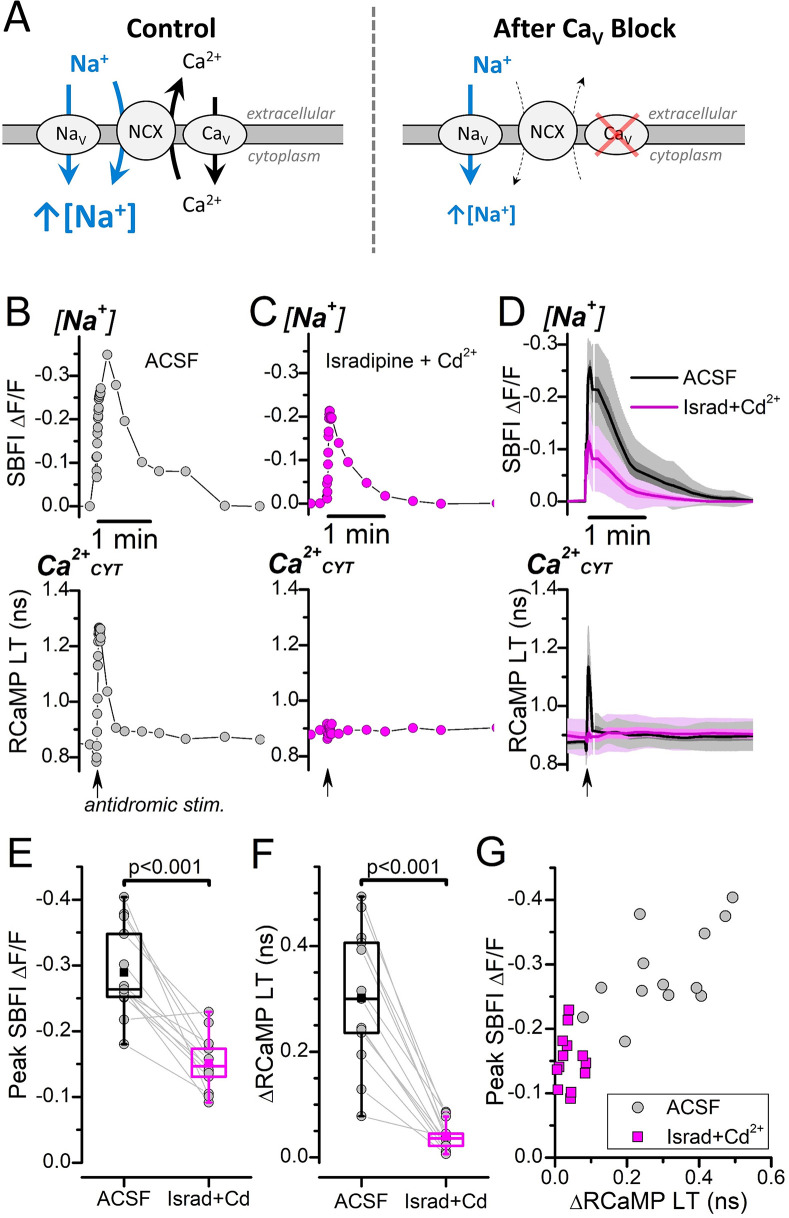
Antidromic stimulation-induced [Na^+^] transients depend strongly on Ca^2+^ entry. (**A**) Cartoon depicting antidromic stimulation-induced Na^+^ and Ca^2+^ fluxes. *Left, Control*: Stimulation in ACSF triggers Na^+^ and Ca^2+^ influx through their respective voltage-gated Na^+^ and Ca^2+^ channels. Following the Ca^2+^_CYT_ elevation, the NCX will import Na^+^ to drive Ca^2+^ extrusion. Both the Na^+^ influx through Na_V_ and through the NCX contribute to the total intracellular [Na^+^] increase (*blue arrows*). *Right, After Ca_V_ Block*: Inhibiting Ca^2+^ influx through Ca_V_ (indicated by the *red X*) prevents Ca^2+^_CYT_ elevation, which strongly reduces Na^+^ influx through the NCX. Abbreviations: voltage-gated Na^+^ channel (Na_V_), voltage-gated Ca^2+^ channel (Ca_V_), Na^+^/Ca^2+^-exchanger (NCX). (**B**) Representative fluorescence traces showing antidromic stimulation-induced transient changes to SBFI ∆F/F (*top*) and RCaMP lifetime (LT) (*bottom*) from a DGC bathed in ACSF with synaptic blockers. The stimulation was delivered at the timepoint indicated by the arrow at the bottom of the RCaMP trace. The SBFI ∆F/F axis is inverted to illustrate an increase to [Na^+^] as an upward deflection. (**C**) Antidromic stimulation-induced transient changes to SBFI ∆F/F and RCaMP LT in the presence of 3 μM isradipine and 20 μM CdCl_2_ for the same DGC as in (**B**). The stimulation was delivered at the timepoint indicated by the arrow below the RCaMP trace. (**D**) Average, interpolated, antidromic stimulation-induced SBFI ∆F/F and RCaMP LT transients (n=13) before (*ACSF, black trace and shading*) and after Ca_V_ block (*Israd+Cd^2+^, magenta trace and shading*). The means are indicated by the solid line, SEM is indicated by the darker shading, and SD is indicated by the lighter shading. (**E**) Paired box plots showing the antidromic stimulation-induced peak transient SBFI ∆F/F amplitudes before (*ACSF, black box*) and after Ca_V_ block (*Israd+Cd^2+^, magenta box*) for the same DGCs as in (**D**). The mean peak SBFI ∆F/F values were: –0.29±0.07 in ACSF and –0.15±0.04 with Israd+Cd^2+^. Statistical significance from a paired sample t-test is indicated. (**F**) Paired box plots showing the ∆RCaMP LT before (*ACSF, black box*) after Ca_V_ block (*Israd+Cd^2+^, magenta box*) for the same DGCs as in (**D**) and (**E**). The mean ∆RCaMP LTs were: 0.30±0.13 ns in ACSF and 0.04±0.03 ns with Israd+Cd^2+^. Statistical significance from a paired sample t-test is indicated. (**G**) Scatterplot of antidromic stimulation-induced transient changes to SBFI ∆F/F against ∆RCaMP LT before (*ACSF, gray circles*) and after Ca_V_ block (*Israd +Cd^2+^, magenta squares*). Data are from the same DGCs as in (**D–F**).

The stimulation of NADH_CYT_ production by Ca^2+^_CYT_ through coupling between forward NCX transport and the Na^+^/K^+^ pump (Figure 3) indicates that Na^+^ influx during forward NCX transport can activate glycolysis. But how does the amount of Na^+^ imported by the NCX to extrude Ca^2+^_CYT_ compare to the total Na^+^ influx induced by neuronal excitation? We investigated this by measuring intracellular [Na^+^] changes during electrical stimulation with and without Ca^2+^ entry (Figure 4A).

**Figure 4—figure supplement 1.**
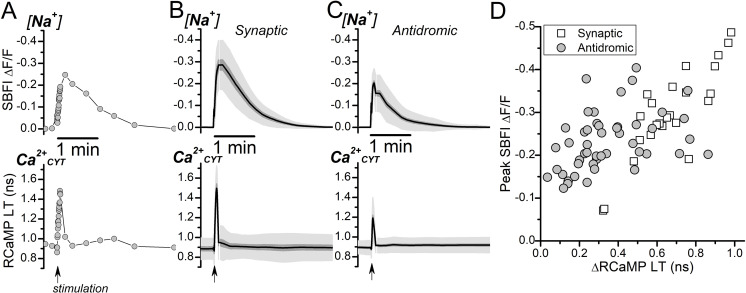
Somatic intracellular [Na^+^] and Ca^2+^_CYT_ transients induced by synaptic or antidromic stimulation. (**A**) Representative fluorescence traces of SBFI ∆F/F (*top*) and RCaMP lifetime (LT; *bottom*) from a DGC when stimulated synaptically in the absence of synaptic blockers (indicated by the arrow along the bottom of the RCaMP trace). The SBFI ∆F/F axis is inverted to illustrate an increase to intracellular [Na^+^] as an upward deflection. (**B–C**) Average, interpolated, transient changes to SBFI ∆F/F (*top*) and RCaMP LT (*bottom*) triggered by synaptic ((**B**), n=24) or antidromic ((**C**), n=48) stimulation. The stimulation was delivered at the time indicated by the arrow at the bottom of the RCaMP trace. The mean is shown as the solid line, SEM is the dark gray shading, and SD is the light gray shading. (**D**) Scatterplot of the peak transient SBFI ∆F/F against the transient ∆RCaMP LT in response to either synaptic stimulation (*Synaptic, open squares*) or antidromic stimulation (*Antidromic, filled circles*) from the same set of DGCs as in (**B**) and (**C**).

We recorded stimulation-induced changes in intracellular [Na^+^] and Ca^2+^_CYT_ simultaneously by loading SBFI (Harootunian et al., 1989), a Na^+^-sensitive fluorescence dye, into RCaMP-expressing DGCs via single-cell electroporation. Representative traces of the SBFI relative fluorescence intensity (∆F/F) and RCaMP LT from a DGC bathed in ACSF show that antidromic stimulation evoked a transient decrease to SBFI ∆F/F (note the inverted y-axis), reflecting an increase to [Na^+^], and a transient increase to RCaMP LT, reflecting an increase to Ca^2+^_CYT_ (Figure 4B). SBFI ∆F/F transients were longer in duration than the RCaMP LT transient, regardless of whether they were evoked by antidromic stimulation or synaptic stimulation in the absence of synaptic blockers (Figure 4 and Figure 4—figure supplement 1), indicating that stimulation-induced intracellular [Na^+^] elevation outlasts Ca^2+^_CYT_ elevation. A scatterplot of the transient changes to SBFI ∆F/F and RCaMP LT shows that intracellular [Na^+^] and Ca^2+^_CYT_ changes are positively correlated when evoked by either stimulation paradigm (Figure 4—figure supplement 1D); while this is consistent with a positive dependence of intracellular [Na^+^] on Ca^2+^_CYT_, a positive correlation between [Na^+^] and Ca^2+^_CYT_ increases would also be observed if neuronal stimulation evokes proportional yet independent changes to both ions.

To directly test if somatic intracellular [Na^+^] changes depend on Ca^2+^ entry, we measured how antidromic stimulation-induced [Na^+^] transients were affected by blocking Ca^2+^ channels (Figure 4A). Blocking voltage-gated Ca^2+^ channels with isradipine and Cd^2+^ (Berjukow et al., 2000; Lansman et al., 1986) attenuated the antidromic stimulation-induced RCaMP LT transient (Figure 4C and D), indicating a strong reduction to Ca^2+^ entry and Ca^2+^_CYT_ elevation. The SBFI ∆F/F transient became smaller after blocking Ca^2+^ entry (Figure 4C and D), demonstrating that stimulation-induced intracellular [Na^+^] changes depend on Ca^2+^_CYT_ elevation; the peak of the SBFI Na^+^ transient (as ∆F/F) was reduced to 54.6±19.3% of the original response and the Ca^2+^ transient (as ∆RCaMP LT) was reduced to 14.5±12.5% (n=13) (Figure 4E and F).

A scatterplot of the antidromic stimulation-induced transient changes to SBFI ∆F/F and ∆RCaMP LT in ACSF or in the presence of isradipine and Cd^2+^ (Figure 4G) shows that blocking Ca^2+^ entry shifts the peak SBFI ∆F/F down and to the left, which indicates that a major component of somatic [Na^+^] changes is dependent on Ca^2+^_CYT_. This means that Na^+^ imported by forward NCX transport in response to Ca^2+^_CYT_ extrusion comprises a substantial proportion of the Na^+^ that enters the soma following stimulation, which provides clarity to the apparent dependence of antidromic stimulation-induced glycolysis activation on Ca^2+^_CYT_ elevation (Díaz-García et al., 2021a): an elevation to Ca^2+^_CYT_ potentiates Na^+^ influx, which ultimately leads to a stronger activation of the Na^+^/K^+^ pump than when Ca^2+^ entry is blocked.

## Discussion

Glycolysis in neurons is transiently increased following stimulation (Díaz-García et al., 2017; Fox et al., 1988; Fox and Raichle, 1986). To understand the mechanism that drives this increase to glycolysis following neuronal electrical activity, we tested the responsiveness of NADH_CYT_ to changes in intracellular Na^+^ and Ca^2+^_CYT,_ which affect energy consumption by ion-ATPases. We have shown in hippocampal DGC somas that NADH_CYT_ is strongly influenced by changes in intracellular Na^+^ and that activation of NADH_CYT_ increases by Ca^2+^_CYT_ is nearly completely dependent on ion transport coupling between the NCX and the Na^+^/K^+^ pump. This means that nearly all of the transient glycolysis increase following a stimulation is a response to an activation of the Na^+^/K^+^ pump to extrude the Na^+^ that enters either through Na^+^ channels or through the NCX to power active Ca^2+^ transport.

### Neuronal glycolysis is strongly influenced by the Na^+^/K^+^ pump, but not by Ca^2+^ pumps

Coupling between glycolysis and Na^+^/K^+^ pump activity has been reported across many tissues and cell types (Andersen and Marmarou, 1992; Balaban and Bader, 1984; Hasin and Barry, 1984; Hellstrand et al., 1984; James et al., 1996; James et al., 1999; Knull, 1978; Lipton and Robacker, 1983; Lynch and Balaban, 1987; Mercer and Dunham, 1981; Minakami et al., 1964; Paul et al., 1979; Paul, 1983; Proverbio and Hoffman, 1977; Weiss and Hiltbrand, 1985; Whittam et al., 1964; Whittam and Ager, 1965). Our lab previously reported a link between the activity of the Na^+^/K^+^ pump α3-isozyme and glycolysis activation in DGCs of acute mouse hippocampal slices: the potentiation to antidromic stimulation-induced NADH_CYT_ production after increasing channel-mediated influx of Na^+^ with α-pompilidotoxin in the absence of external Ca^2+^ (i.e., when both Na^+^ influx through the NCX and Ca^2+^ influx through channels are blocked) could be reversed by strophanthidin at low micromolar concentrations (Díaz-García et al., 2021a). We have shown here in DGCs that NADH_CYT_ production decreases with depletion of Na^+^ and increases with repletion of Na^+^ (Figure 1), which strongly suggests that the rate of neuronal glycolysis mirrors changes in intracellular [Na^+^] and in the activity of the Na^+^/K^+^ pump. Moreover, DGCs could not withstand complete Na^+^/K^+^ pump inhibition when bathed in ACSF (Figure 3—figure supplement 1), meaning that Na^+^/K^+^ pump activity is essential to counteract Na^+^ leak and/or influx through Na^+^-coupled transporters even when neurons are at rest.

The small NADH:NAD^+^ increase produced by removing only external Ca^2+^ that we observed during our previous studies (Díaz-García et al., 2021a) is also consistent with a predominant influence of Na^+^ on glycolysis: since Na^+^ conductance through the NALCN (Na^+^-leak channel, non-selective), which regulates DGC resting membrane potential and excitability (S.-Y. Lee et al., 2019), is higher in the absence of external Ca^2+^ (Kschonsak et al., 2020; Lu et al., 2010), removing external Ca^2+^ likely elevates Na^+^ influx through the NALCN and increases Na^+^/K^+^ pump activity.

The sensitivity of glycolysis to Ca^2+^ pump activity is less clear. The small increases to the antidromic stimulation-induced ∆Peredox/∆RCaMP ratio after applying inhibitors that target SERCA (thapsigargin; Figure 1—figure supplement 2) or PMCA (E6-berbamine or calmidazolium; Brini et al., 2013; Díaz-García et al., 2021a) do suggest some involvement of Ca^2+^ pumps in regulating stimulation-induced Ca^2+^_CYT_ increases, but our observations that NADH_CYT_ was not increased by the elevation of Ca^2+^_CYT_ alone, either by reverse NCX transport during ion substitution or Ca^2+^ puffs in either zero external Na^+^ or 5 mM ouabain, indicate that ATP consumption associated with the direct pumping of Ca^2+^ does not have a measurable effect on glycolysis (Figures 1—3, and Figure 1—figure supplement 1). This may indicate that Ca^2+^ pumps are either not closely coupled to glycolytic enzymes in DGCs or that Ca^2+^ pump density is small with respect to Na^+^/K^+^ pumps. It appears that the density of Na^+^/K^+^ pumps outweighs the density of Ca^2+^ pumps in hippocampus since hippocampal homogenates have ouabain-sensitive ATPase activity ~2- to 3-fold larger than Ca^2+^-sensitive ATPase activity (Gutierres et al., 2014; Spohr et al., 2022; Teixeira et al., 2020). Even if we assume that Ca^2+^ pumps and Na^+^/K^+^ pumps are coupled to glycolysis equally, the lower density of Ca^2+^ pumps would make their glycolytic (and total cellular) ATP consumption less than the ATP consumed by Na^+^/K^+^ pumps.

Ca^2+^ pumps have an apparent K_0.5_ for Ca^2+^ activation of ~0.1 μM and relatively slow maximal turnover rates of ~100 s^–1^ (characteristic of ion pumps; Glitsch, 2001; Meyer et al., 2017), meaning that they are well suited for tuning the resting Ca^2+^_CYT_ but not for regulating large dynamic Ca^2+^_CYT_ increases (Caroni and Carafoli, 1981). It is likely that Ca^2+^ pump transport capacity would saturate quickly in response to the 0.3–1 μM increases in Ca^2+^ during some stimulations. If an increase to Ca^2+^ pump activity was the predominant driver of NADH_CYT_ production, the saturation of their Ca^2+^ transport during larger Ca^2+^_CYT_ increases would likely cause a ‘rounding off’ of the Peredox LT change for stimulations where RCaMP LT changes are >0.5 ns; but the near linearity of the ∆Peredox-∆RCaMP relationship for RCaMP LT changes of 0.05–1 ns (Díaz-García et al., 2017) argues against such a scenario.

### The Ca^2+^_CYT_-dependence of aerobic glycolysis activation arises from ion transport coupling between the Na^+^/Ca^2+^-exchanger and the Na^+^/K^+^ pump

Our observations here that glycolysis activation in DGCs is strongly coupled to the activity of the Na^+^/K^+^ pump but not Ca^2+^ pumps was seemingly at odds with the ~70% reduction to antidromic stimulation-induced NADH_CYT_ production by blocking Ca^2+^_CYT_ elevation (Díaz-García et al., 2021a). However, the reduction to NADH_CYT_ production is fully explained by the involvement of forward NCX transport in DGC Ca^2+^_CYT_ regulation (Figure 1F; Lee et al., 2012) and by the close coupling between the ion transport of the NCX and the Na^+^/K^+^ pump.

The NCX has an apparent K_0.5_ for internal Ca^2+^ of ~1 μM (Blaustein and Santiago, 1977; Collins et al., 1992; Miura and Kimura, 1989) and a transport rate 10–30 times faster than the slow turnover rates of ATPases (Blaustein and Lederer, 1999; Caroni et al., 1980). This means that NCX transport activity would be near the lower end of its total capacity when neurons are at rest (when Ca^2+^_CYT_ is ~100 nM) but could rapidly respond to stimulation-induced Ca^2+^_CYT_ increases and exchange it for Na^+^. Unfortunately, most NCX inhibitors are unspecific or do not block the forward transport mode (Iwamoto et al., 2004; Iwamoto and Kita, 2006; Secondo et al., 2015; Sharikabad et al., 1997), so the most effective way to test the involvement of forward NCX transport in glycolysis activation is to replace external Na^+^ with an inert ion that cannot be transported by the NCX (i.e., choline).

The transient NADH_CYT_ production evoked by Ca^2+^ puffs in 40 mM Na^+^ and 0 Ca^2+^ was eliminated when external Na^+^ was completely removed (i.e., replaced with choline) or when ouabain was added (Figure 3), meaning that both forward NCX transport and Na^+^/K^+^ pump activity are required for a Ca^2+^_CYT_ elevation to measurably activate glycolysis. This unequivocally demonstrates that the exchange of Ca^2+^_CYT_ for Na^+^ by the NCX is closely coupled to Na^+^ extrusion by the Na^+^/K^+^ pump, and that this coupling is tightly associated with the activation of glycolysis. Coupling between the NCX and Na^+^/K^+^ pump within discrete microdomains has also been reported in cardiac tissue (Mohler et al., 2005; Zhang et al., 2009; Zhang et al., 2011).

Strong coupling between the NCX, the Na^+^/K^+^ pump, and glycolysis activation can also explain two of our other observations: First, it can explain why the stimulation-induced ∆Peredox/∆RCaMP ratio is increased by inhibition of SERCA or PMCA. Blocking either of these Ca^2+^ pumps likely increases the Ca^2+^ that is exported by the NCX, which would amplify the associated Na^+^ influx through the NCX and accelerate the ATP consumption by the Na^+^/K^+^ pump to extrude Na^+^. Second, it can explain why NADH_CYT_ production was decreased when Ca^2+^_CYT_ was elevated by reverse NCX transport (Figure 1 and Figure 1—figure supplement 1). For reverse NCX transport to import 1 Ca^2+^ from the extracellular environment, the NCX must export 3 Na^+^ (Figure 1A). This means that an increase to Ca^2+^_CYT_ would occur at the same time as a decrease to intracellular [Na^+^], which would decrease the activity of the Na^+^/K^+^ pump and, consequently, decrease the rate of glycolysis and its associated NADH_CYT_ production. The decrease to cytosolic NADH:NAD^+^ from reverse NCX transport further supports the strong association between the energy consumed to extrude Na^+^ and glycolysis and implies that even decreasing the Na^+^/K^+^ pump activity from an already reduced steady-state level of activity (due to the depletion of intracellular [Na^+^] from prior application of the 0 Na 0 Ca solution) is more influential on the rate of glycolysis than any putative strong activation of Ca^2+^ pumps by a Ca^2+^_CYT_ increase.

The ∆Peredox/∆RCaMP ratio evoked by Ca^2+^ puffs in 40 mM Na^+^ and 0 Ca^2+^ (~0.1, Figure 3E) is ~40–50% of the ∆Peredox/∆RCaMP ratio evoked by electrical stimulation in ACSF (~0.2–0.25) (Díaz-García et al., 2017; Díaz-García et al., 2021a), meaning that the peak activation of glycolysis relative to the Ca^2+^_CYT_ change is smaller when evoked by Ca^2+^ puffs compared to electrical stimulation. This could be due to the absence of channel-mediated Na^+^ entry during Ca^2+^ puffs compared to electrical stimulation or to the reduced external [Na^+^] in Ca^2+^ puff experiments (40 mM) compared to the external [Na^+^] during electrical stimulation in ACSF (147 mM), which would slow down the kinetics of Ca^2+^_CYT_-dependent Na^+^ influx through forward NCX transport, consequently leading to a less potent activation of the Na^+^/K^+^ pump; both the quantity and the rate of Na^+^ influx are likely to be important determinants of the NADH_CYT_ transient amplitude and time course.

Glycolysis does not appear to be influenced by intracellular [Na^+^] per se, but rather by the downstream effect of [Na^+^] on Na^+^/K^+^ pump activity. If glycolytic enzymes could be directly stimulated by an increase to [Na^+^], then complete inhibition of the Na^+^/K^+^ pump should have facilitated stronger NADH_CYT_ responses. But this was not the case, as NADH_CYT_ responses evoked by Ca^2+^ puffs were strongly attenuated in the presence of 5 mM ouabain (Figure 3), a condition that allows for Na^+^ influx through the NCX but prevents Na^+^ extrusion through the Na^+^/K^+^ pump. In contrast to the strong attenuation by 5 mM ouabain, the increase to the NADH_CYT_ transients evoked by synaptic electrical stimulation after applying 5 μM ouabain in ACSF (Figure 3—figure supplement 2) is not as straightforward and cannot be attributed solely to an inhibition of the Na^+^/K^+^ pump. First, 5 μM ouabain does not substantially inhibit rodent α1-pumps (Marks and Seeds, 1978; Sweadner, 1979); we know that 5 μM ouabain only causes partial Na^+^/K^+^ pump inhibition in our acute slice preparation from the mouse because it does not cause Ca^2+^_CYT_ overload (unlike 5 mM ouabain in ACSF), which means that the plasma membrane Na^+^ gradient in the presence of 5 μM ouabain remains sufficient to prevent a reversal of NCX transport. Second, the stimulation-induced Ca^2+^_CYT_ transients were increased, which is, perhaps, reflective of an effect on synaptic release.

We think that the absence of strong NADH_CYT_ responses to Ca^2+^_CYT_ increases when Na^+^ is unavailable for exchange via NCX (Figure 1 and Figure 3) is quite informative on how both ions influence glycolysis activation, even though it is true that the rapid ionic changes that occur through channels during neuronal activity following electrical stimulation might obey somewhat different rules than the slower ionic changes we are able to make by driving ion fluxes through a secondary ion transporter. Since we have determined that NADH_CYT_ production is strongly increased by an influx of Na^+^ but not increased by large increases in Ca^2+^_CYT_ (Figures 1—3) it is logical to infer that a transient increase to NADH_CYT_ production following electrical stimulation is primarily coupled to the energy consumed to directly pump Na^+^ rather than to directly pump Ca^2+^. The amplitudes of RCaMP LT increases that we achieve with both the ion substitution experiments (Figure 1) and the Ca^2+^ puff experiments (Figure 3) are also similar to the typical amplitude of RCaMP LT increase when evoked by electrical stimulation (Figure 4 and Díaz-García et al., 2017; Díaz-García et al., 2021a), meaning that Ca^2+^_CYT_ is increased to similar levels by all experimental paradigms regardless of their site of entry (i.e., channels or NCX).

### Na^+^ entry via the Na^+^/Ca^2+^-exchanger contributes substantially to total Na^+^ entry in the soma

It seemed that stimulation-induced Na^+^ influx via the NCX could be substantial given that a transient Ca^2+^_CYT_ increase could only activate glycolysis if both forward NCX transport and the Na^+^/K^+^ pump were active (Figure 3) and that blocking Ca^2+^_CYT_ strongly reduces the NADH_CYT_ response (Díaz-García et al., 2021a). The ~50% reduction to the stimulation-induced SBFI ∆F/F peak amplitudes after blocking Ca^2+^ entry confirmed that Na^+^ influx via forward NCX transport contributes greatly to the total Na^+^ influx in the soma (Figure 4). We think it is unlikely that the reduction to the [Na^+^] transient after blocking Ca^2+^ entry resulted from less Na^+^ entering through voltage-gated Ca^2+^ channels, since Na^+^ permeation through these channels is blocked by external Ca^2+^ with a K_d_ of ~1 µM (Almers and McCleskey, 1984; Tsien et al., 1987).

It is interesting to note that the positive relationship between the amplitudes of stimulation-induced transient increases to NADH_CYT_ and Ca^2+^_CYT_ appears to intercept the origin (cf. Figure 2; Díaz-García et al., 2017) while the relationship between the amplitudes of transient intracellular [Na^+^] and Ca^2+^_CYT_ increases does not (Figure 4—figure supplement 1). Perhaps weaker stimulations that evoke small increases to intracellular [Na^+^] without a measurable increase to Ca^2+^_CYT_ have only very weak effects on glycolysis: it could be that smaller [Na^+^] increases have proportionally more clearance by diffusion through the cytoplasm (Hodgkin and Keynes, 1956; Pusch and Neher, 1988) or that the energy needed to regulate smaller [Na^+^] increases (without sustained Na^+^ influx through forward NCX transport) is mostly derived from ATP buffering systems known to compartmentalize near sites of high ATP turnover (Kültz and Somero, 1995; Lange et al., 2002; Ovádi and Saks, 2004; Wallimann et al., 1992), which could subsequently replenish their energy storage pools without large steady-state changes to glycolytic flux. Directly evaluating the role of these ATP buffering enzymes, that is, creatine kinase and adenylate kinase, in shaping the stimulation-induced transient glycolysis activation in neurons will be a key area for future exploration.

### Estimation of neuronal stimulation-induced changes to intracellular [Na^+^] and Na^+^/K^+^ pump activity

SBFI calibration curves in rodent hippocampal neurons (Baeza-Lehnert et al., 2019; Diarra et al., 2001; Gerkau et al., 2019; Meier et al., 2006; Rose et al., 1999) reported apparent Na^+^ K_d_ values of 18–42 mM Na^+^. However, most were performed with ouabain concentrations of only 50–100 μM, well below the millimolar concentrations needed to fully inhibit the rodent Na^+^/K^+^ pump α1 isozyme under physiological conditions; the relatively high ouabain-resistance of rodent α1 pumps would also be strongly increased in calibration solutions that substitute external Na^+^ with high concentrations of K^+^ since external K^+^ competes with binding of the inhibitor to the pump’s externally accessible E2 conformation (Hansen and Skou, 1973; Kanai et al., 2021).

Despite the limitations of these in situ SBFI calibrations in rodent neurons, we use Rose et al.’s calibration of SBFI ∆F/F excited at 790 nm in brain slices with a Na^+^ K_d_ of 26 mM (Rose et al., 1999) to *estimate* the stimulation-induced [Na^+^] changes in our experiments (which reflect the net movement of Na^+^ through influx and extrusion pathways). Our estimations assume a baseline intracellular [Na^+^] of 13 mM, which is the reported value for hippocampal slice CA1 neurons (Mondragão et al., 2016) and nearly identical to the K_0.5_ for activation of internal Na^+^-dependent, ATP-induced current produced by α1β1 Na^+^/K^+^ pumps (which likely set baseline neuronal internal [Na^+^]; Azarias et al., 2013; Blanco, 2005; Blanco and Mercer, 1998) in excised patches when internal Na^+^ is substituted with K^+^ (Meyer et al., 2017; Meyer et al., 2019; Meyer et al., 2020).

The mean peak amplitude of antidromic stimulation-induced SBFI ∆F/F transients evoked in ACSF was –25.6±4.6% (Figure 4), reflecting an average transient [Na^+^] increase of ~25 mM from baseline. How would this intracellular [Na^+^] change affect neuronal Na^+^/K^+^ pump activity? Neurons express the α1 and α3 isoforms of the Na^+^/K^+^ pump (Blanco and Mercer, 1998). Under physiological conditions, both α1 and α3 pumps are rate limited by the binding of internal Na^+^ since their K_0.5,Na_^+^ is similar to, or above, physiological intracellular [Na^+^] while their K_0.5,K_^+^ is below physiological extracellular [K^+^]. Activation of Na^+^/K^+^ pumps by internal Na^+^ is also highly cooperative (Hill coefficient of ~3, corresponding to three transported Na^+^ Crambert et al., 2000; Hasler et al., 1998; Meyer et al., 2017; Meyer et al., 2019; Meyer et al., 2020). Based on the reported curves of Na^+^/K^+^ pump activation by internal Na^+^ (Crambert et al., 2000; Meyer et al., 2019), a 25 mM increase to neuronal [Na^+^] from a baseline of 13 mM would likely increase peak α1β1 activity ~2-fold (to ~95% of total activity) and α3 activity ~9-fold (to ~60% of total activity).

Blocking Ca^2+^ entry reduced the antidromic stimulation-induced peak SBFI ∆F/F to –11.5±5.5% (Figure 4), a ~55% decrease from the transient in ACSF that corresponds nonlinearly to a reduction of the average [Na^+^] increase from ~25 to ~8 mM. The estimated 8 mM stimulation-induced increase to internal [Na^+^] when Ca^2+^ entry is inhibited (i.e., when Na^+^ influx via forward NCX transport is reduced) would increase α1β1 activity ~1.5-fold (to ~80% of total activity) and α3 activity ~3-fold (to ~25% of total activity).

It must be noted that the proportional influence of NCX-mediated Na^+^ entry on glycolysis activation could be dependent on the stimulation paradigm. Antidromic stimulation in the presence of synaptic blockers does not involve entry of Na^+^ through NMDA or AMPA receptors. This means that most channel-mediated Na^+^ influx that occurs from this stimulation method is through voltage-gated channels, which, in DGCs, are highly localized to the axon initial segment with weak, if any, staining in the soma (Kole et al., 2008; Kress et al., 2008; Kress et al., 2010). Thus, it is not unexpected that Na^+^ influx via forward NCX transport has such a prominent effect on the antidromic stimulation-induced glycolysis activation in the soma. Soma SBFI transients evoked by either antidromic or synaptic stimulation could reach similar peak amplitudes (Figure 4—figure supplement 1), but the contribution of Na^+^ influx through forward NCX transport to synaptically evoked [Na^+^] changes could not be tested because blocking voltage-gated Ca^2+^ entry would prevent synaptic vesicle release.

NCX involvement in glycolysis activation could also vary in different neuronal compartments. Reported SBFI transient measurements indicate that antidromic stimulation-induced [Na^+^] changes in the soma are typically smaller than transients in the dendrites (Fleidervish et al., 2010; Kole et al., 2008). Measuring the ion and metabolite dynamics in dendrites will be an important step for understanding whether the mechanism of glycolysis activation in dendrites is similar to or different than the mechanism in somas.

### On the mechanism of neuronal stimulation-induced aerobic glycolysis activation in somas

Based on our observations here and previously (Díaz-García et al., 2017; Díaz-García et al., 2021a) regarding neuronal stimulation-induced [Na^+^], Ca^2+^_CYT_, and NADH_CYT_ dynamics in the soma, we can begin to compile a more comprehensive understanding of the mechanism underlying transient aerobic glycolysis activation in response to neuronal excitation.

Neuronal stimulation immediately increases both intracellular [Na^+^] and Ca^2+^_CYT_. The transient Ca^2+^_CYT_ elevation lasts at most a few seconds (slightly longer than the duration of the stimulus; RCaMP1h has a t_1/2_ decay of 410 ms; Akerboom et al., 2013) before returning to baseline due to buffering by cytosolic proteins and removal from the cytosol. This means that any transient activation of Ca^2+^ pumps by Ca^2+^_CYT_ would likely only be a few seconds in duration before returning to steady state. In any case, we know that this produces no measurable activation of glycolysis since similar Ca^2+^_CYT_ transients evoked by Ca^2+^ puffs in the absence of external Na^+^ or presence of ouabain were not associated with NADH_CYT_ increases. It is also clear that Ca^2+^_CYT_ is rapidly exported across the plasma membrane by forward NCX transport, which loads the neuron with Na^+^. Na^+^ is not buffered in the cytoplasm and is actively extruded back across the plasma membrane exclusively by the Na^+^/K^+^ pump. The transient intracellular [Na^+^] increase lasts for a minute or more (longer than the Ca^2+^_CYT_ transient), during which an increase to the activity of Na^+^/K^+^ pumps would be sustained. Both the sensitivity of NADH_CYT_ production to Na^+^ and the ablation of Ca^2+^_CYT_-evoked NADH_CYT_ transients by inhibition of either forward NCX transport or the Na^+^/K^+^ pump demonstrate that pumping Na^+^ is the predominant activity that drives the transient activation of aerobic glycolysis.

Stimulation also triggers a rapid increase to Ca^2+^_MITO_ via MCU that can last several minutes (Díaz-García et al., 2021a) and requires Na^+^ in order to return to the baseline (Figure 1—figure supplement 3). The requirement of Na^+^ for Ca^2+^_MITO_ extrusion likely indicates a Na^+^-coupled Ca^2+^ efflux mechanism, as reported in other cell types (Palty and Sekler, 2012; Parpura et al., 2016; Saotome et al., 2005). Interestingly, the attenuation of Ca^2+^_MITO_ elevation from MCU knockdown reduces the stimulation-induced cytosolic ∆Peredox/∆RCaMP by ~50% (Díaz-García et al., 2021a), which is consistent with Ca^2+^_MITO_ extrusion stimulating glycolysis. The extent to which the energetic burden of Ca^2+^_MITO_ extrusion is placed onto glycolysis, and, more specifically, the Na^+^/K^+^ pump, or elsewhere will be an important area for future exploration.

Given the close coupling of the Na^+^/K^+^ pump to neuronal glycolysis, it will be exciting to measure how changes in Na^+^/K^+^ pump activity affect the flux of other glycolytic metabolites using biosensors that report lactate (Koveal et al., 2022; San Martín et al., 2013), pyruvate (San Martín et al., 2014), and glucose (Díaz-García et al., 2019), or how changes in Na^+^/K^+^ pump activity affect cellular energy status (i.e., [ATP] [Imamura et al., 2009] and ATP:ADP [Tantama et al., 2013]). These investigations will ultimately provide a more complete picture of the relationship between ion homeostasis and metabolism.

## Materials and methods

**Key resources table:** 

Reagent type (species) or resource	Designation	Source or reference	Identifiers	Additional information
Strain, strain background (*Mus musculus*, M and F)	C57BL/6NCrl	Charles River	RRID:IMSR_CRL:27	
Recombinant DNA reagent	AAV.CAG.Peredox.WPRE.SV40	Mongeon et al., 2016		Addgene #73807
Recombinant DNA reagent	AAV.hSyn.RCaMP1h.WPRE.SV40	Akerboom et al., 2013		
Recombinant DNA reagent	AAV.hSyn.mito-RCaMP1h.WPRE.SV40	Díaz-García et al., 2021a		
Chemical compound, drug	Isradipine	Abcam	Cat: ab120142, CAS: 75695-93-1	
Chemical compound, drug	NBQX (6-Nitro-7- sulfamoylbenzo [f]quinoxaline-2,3-dione, Disodium Salt)	Toronto Research Chemicals	Cat: N550005, CAS: 479347-86-9	
Chemical compound, drug	D-AP5 (D-(-)–2-Amino- 5-phosphonopentanoic acid)	Abcam	Cat: ab120003, CAS: 79055-68-8	
Chemical compound, drug	Picrotoxin	Sigma-Aldrich	Cat: P1675 CAS: 124-87-8	
Chemical compound, drug	CdCl_2_	Sigma-Aldrich	Cat: 202908, CAS: 10108-64-2	
Chemical compound, drug	Poly-L-lysine	Sigma-Aldrich	Cat: P4832	
Chemical compound, drug	Aminooxyacetate (O-(carboxymethyl)hydroxylamine hemihydrate)	Sigma-Aldrich	Cat: C13408 CAS: 2921-14-4	
Chemical compound, drug	Pyruvic acid	Sigma-Aldrich	Cat: 107360, CAS: 127-17-3	
Chemical compound, drug	Thapsigargin	Santa Cruz Biotechnology	Cat: sc-24017A, CAS: 67526-95-8	
Chemical compound, drug	Ouabain	Sigma-Aldrich	Cat: O3125, CAS: 11018-89-6	
Chemical compound, drug	SBFI K^+^ salt (fluorescent dye)	Ion Biosciences	Cat: 2022B	
Other	Glass capillaries, Borosilicate, standard wall, no filament, 4 in., O.D. 1.5 mm	WPI	Cat: 1B150-4	For microelectrodes and pipettes
Other	Glass coverslips, 12 mm circle No.1	VWR	Cat: 48366–251	For brain slice handling

### Animals

Experiments were performed using male and female wild-type mice (C57BL/6NCrl, Charles River Laboratories), which were housed in a barrier facility in individually ventilated cages with ad libitum access to standard chow (PicoLab 5053). All experiments followed approved IACUC protocols and the NIH Guide for the Care and Use of Laboratory Animals and Animal Welfare Act. All procedures were approved by the Harvard Medical Area Standing Committee on Animals.

### Viral vectors

DNA constructs encoding Peredox, RCaMP, or mito-RCaMP biosensors were packaged into adeno-associated virus (AAV) vectors using either the Penn Vector Core at University of Pennsylvania or the Viral Core Facility at Children’s Hospital in Boston, MA. AAV vectors encoding RCaMP were also produced in our laboratory, as previously described (Kimura et al., 2019). The AAV8 serotype was used for Peredox and AAV9 for RCaMP and mito-RCaMP. AAVs were aliquoted and stored at –80°C.

### Intracranial injections

Postnatal day 1 or 2, mice were injected intracranially with AAV to express biosensors in the hippocampus (Díaz-García et al., 2021b). Mice were cryoanesthetized and injected with 150 nL AAV using an UltraMicroPump III (WPI, Sarasota, FL) microinjector in two locations of each hemisphere at the following coordinates relative to lambda: (1) 0 mm anterior-posterior; ±1.9 mm medial-lateral; –2.0 mm dorsal-ventral; and (2) 0 mm anterior-posterior; ±2.0 mm medial-lateral; –2.3 mm dorsal-ventral. Injected pups were placed on a heating pad and allowed to recover before being returned to their cages and were administered daily ketoprofen (10 mg/kg) subcutaneously for up to 3 days.

### Hippocampal slice preparation

Injected mice between 14 and 24 days old were anesthetized with isoflurane, decapitated, and the brain was removed into ice-cold slicing solution containing (in mM) 87 NaCl, 2.5 KCl, 1.25 NaH_2_PO_4_, 25 NaHCO_3_, 75 sucrose, 25 D-glucose, 0.5 CaCl_2_, and 7 MgCl_2_ (~335 mOsm/kg) and bubbled with 95% O_2_ and 5% CO_2_. The brain was glued by the dorsal side, embedded into 2% agarose in phosphate-buffered saline, and submerged in a chamber with ice-cold slicing solution. Horizontal 275 μm brain slices were cut using a compresstome (VF-310-0Z, Precisionary) and immediately transferred to a chamber with ACSF containing (in mM) 120 NaCl, 2.5 KCl, 1 NaH_2_PO_4_, 26 NaHCO_3_, 10 D-glucose, 2 CaCl_2_, and 1 MgCl_2_ (~290 mOsm/kg) that was warmed to 36°C and bubbled with 95% O_2_ and 5% CO_2_. The slices rested on a mesh bottom to adequately perfuse both sides of the tissue. After 35 min, the chamber was cooled to room temperature and the brain slices therein were used for the next 4 hr.

### Recording solutions and pharmacology

The brain slices were adhered to glass coverslips coated with poly-L-lysine (P4832, Sigma-Aldrich), placed in a bath chamber mounted to the microscope, and superfused with solutions maintained at 33–34°C and bubbled with 95% O_2_ and 5% CO_2_ at a rate of 5 mL/min. All solutions were ~290 mOsm/kg and contained 25 μM D-AP5, 5 μM NBQX, and 100 μM picrotoxin (unless otherwise specified) to block synaptic transmission. Ca^2+^-free solution contained (in mM) 120 NaCl, 2.5 KCl, 1 NaH_2_PO_4_, 26 NaHCO_3_, 10 D-glucose, and 4.1 MgCl_2_. Na^+^- and Ca^2+^-free solution contained (in mM) 120 Choline-Cl, 1.5 KCl, 1 KH_2_PO_4_, 26 Choline-HCO_3_, 10 D-glucose, and 4.1 MgCl_2_. 1 mM EGTA was added to the Ca^2+^-free solutions from a 0.5 M stock.

For Ca^2+^-puff experiments, the 40 mM Na^+^ and Ca^2+^-free solution was obtained by mixing Ca^2+^-free solution with Na^+^- and Ca^2+^-free solution; all solutions for those experiments also contained 0.1 mM EGTA.

Thapsigargin (1 μM) was added from a 10 mM stock in DMSO. Aminooxyacetate (AOA, 10 mM) was added from a 2 M stock in water. Pyruvate (1.5 mM) was added from a 1 M aqueous stock, pH 7.3 with *N*-methyl-D-glucamine. Ouabain was either directly dissolved (for 2–5 mM) prior to recording or added from a 5 mM stock in water (for 5 μM). Isradipine (3 μM) was added from a 50 mM DMSO stock. CdCl_2_ (20 μM) was added from a 100 mM stock in water.

### Electrical stimulation

Stimulation trains (of 100 μs pulse width) were delivered as previously described (Díaz-García et al., 2017; Díaz-García et al., 2021a) using a concentric bipolar electrode (FHC, Bowdoin, ME) mounted on a motorized micromanipulator (Burleigh PCS-6000, ThorLabs, Sterling, VA) and connected to an A360 stimulus isolation unit (WPI, Sarasota, FL). DGCs were stimulated antidromically (100 pulses, 50 Hz, 750–1500 μA) by placing the electrode in the hippocampal hilus, or synaptically (60 pulses, 20 Hz, 100–500 μA) by placing the electrode in the molecular layer.

### Ca^2+^ puffs

Borosilicate glass pipettes (with a tip diameter of 5 μm and resistance of 1 MΩ when filled with ACSF) were fabricated on a micropipette puller (P-97 Flaming/Brown, Sutter Instruments), backfilled with 100–250 mM CaCl_2_ and placed into a pipette holder mounted on a motorized micromanipulator with a closed pressure system connected to a 1 mL syringe. The pipette tip was placed adjacent to groups of DGCs expressing Peredox and RCaMP biosensors. Pressure delivery was controlled by a solenoid valve (NResearch) in series with the syringe line that was triggered by a digital/analog actuator (NTE Electronics). Positive pressure was applied using the syringe, and Ca^2+^ puffs were delivered by opening the solenoid for 0.5–5 s beginning 300 ms after the start of image acquisition.

### Single-cell electroporation of SBFI

Sodium-binding benzofuran isophthalate (SBFI) is a fluorescent Na^+^-indicator (Harootunian et al., 1989). We loaded the SBFI K^+^ salt (Ion Biosciences, San Marcos, TX) version into DGCs using single-cell electroporation methods derived from Nevian and Helmchen, 2007; we chose this method rather than loading by patch pipette to avoid diluting cytoplasmic metabolites and soluble expressed biosensor, and also to avoid potentially toxic effects on the slice from applying Pluronic-F127 and DMSO to load the membrane-permeant SBFI-acetoxymethyl ester version.

Borosilicate glass pipettes were fabricated (with a tip diameter of 1 μm and resistance of 3 MΩ when filled with ACSF), loaded with 4 mM SBFI-K^+^ in distilled H_2_O, and placed onto a pipette holder with a closed pressure system mounted on a motorized micromanipulator. The SBFI solution was contacted by a chlorided silver wire connected to an A360 stimulus isolation unit, and an Ag-AgCl reference electrode was placed in the bath chamber and connected to the same stimulation device. The pipette tip was placed directly next to the soma of an RCaMP-expressing DGC. No positive pressure was applied. A single 10 ms pulse of negative 1.0–1.2 μA loaded the DGC with SBFI. Recovery of the membrane potential from these electroporation pulses takes ~1–2 min (Nevian and Helmchen, 2007). We waited ≥15 min after the electroporation to start the image acquisition and electrically stimulate the SBFI-loaded, RCaMP-expressing DGCs.

The intracellular [dye] from electroporation is estimated to be ~20% of the pipette concentration (Nevian and Helmchen, 2007). Thus, the intracellular [Na^+^] will not be substantially buffered by the ~0.5–1 mM [SBFI] in our recordings (Fleidervish et al., 2010; Mondragão et al., 2016), meaning that intracellular [Na^+^] is set by cellular mechanisms and SBFI merely responds to changes in [Na^+^]. Also, the complexation of Na^+^ with crown ether molecules (the Na^+^-sensitive moiety of SBFI) is nearly diffusion-controlled, with very rapid rates of formation and dissociation (Adamic et al., 1986; Liesegang et al., 1977). This means that the response time of SBFI fluorescence changes should be fast compared to almost all neuronal processes.

### Two-photon fluorescence imaging

Fluorescence imaging data were acquired using a Thorlabs Bergamo II microscope (Thorlabs Imaging Systems, Sterling, VA) equipped with an Olympus LUMPLFLN 60×/W (NA 1.0) objective lens, hybrid photodetectors R11322U-40 (Hamamatsu Photonics, Shizuoka, Japan), and a Chameleon Vision-S tunable Ti-Sapphire mode-locked laser (80 MHz,~75 fs pulses; Coherent, Santa Clara, CA). The excitation wavelength was 790 nm. Fluorescence emission light from the Peredox and RCaMP biosensors was split with an FF562-Di03 dichroic mirror and bandpass filtered for green (FF01-525/50) and red (FF01-641/75) light; a red 670/50 bandpass filter was used for experiments where SBFI and RCaMP were multiplexed. The photodetector and laser sync signals were preamplified and then digitized at 1.25 GHz using a field-programmable gate array board (PC720 with FMC125 and FMC122 modules, 4DSP, Austin, TX). A modified version of the ScanImage software written in Matlab (Pologruto et al., 2003) (provided by B. Sabatini and modified by G.Y.) controlled the laser, microscope, and image acquisition (128×128 pixels, scanning rate of 2 ms per line).

Time-correlated single-photon counting was performed using laboratory-built firmware and software to determine the arrival time of each photon relative to the laser pulse. The fluorescence LT was determined from a nonlinear least-squares fit to the photon arrival histograms in Matlab (Mathworks, Natick, MA) convolved with a Gaussian for the impulse response function (Yasuda et al., 2006). Fluorescence intensity was determined from the total photon counts.

#### Data analysis

Fluorescence images were analyzed offline using Matlab R2014b software. Regions of interest (ROIs) were defined around individual DGC somas and photon statistics were calculated for all pixels within the ROI. LT values were calculated by fitting the photon arrival histograms with a biexponential decay function (convolved with a Gaussian for the impulse response function [Yasuda et al., 2006] up to 8 ns after the peak photon arrival time). The time constant of this fit is denoted as the ‘tau8‘ value (Díaz-García et al., 2019); using tau8 values minimizes both the fit variability (by restricting the averaging to the approximate time window of the actual data) and the variability between the LTs recorded on different experimental setups with different data acquisition windows.

RCaMP signals were unmixed from Peredox as previously described (Díaz-García et al., 2017; Díaz-García et al., 2021a). RCaMP signals were unmixed from SBFI bleedthrough into the red 670/50 bandpass filter following previous methods (Díaz-García et al., 2017) but using an unmixing ratio of 0.053, since the red channel photon counts from SBFI-loaded DGCs (not expressing RCaMP) were (mean ± SD) 5.3±1.2% (n=42) of the green channel photon counts. Stimulation-induced SBFI intensity recordings were analyzed using Origin 8.1 (OriginLab, Northampton, MA) and Python 3 (https://www.anaconda.com/, RRID – SRC:008394, Numpy and Pandas libraries). Individual transient fluorescence intensity traces were baseline subtracted and the peak ∆F/F was determined as the minimum intensity value.

Average time traces of transient fluorescence LT or intensity changes were created using Python. Data acquisition times for individual fluorescence traces were binned to the nearest second for data acquired before and after the stimulation event (which were acquired every 10–60 s) or the nearest 10 ms for data acquired during the stimulation (which were acquired every ~250 ms). Traces from all stimulation-induced transients in the data set were interpolated and merged onto a single time axis, from which the means, standard deviations, and standard error of the means were determined.

Statistical analysis was performed using Origin 8.1 software. Data sets were tested for normality (*α*=0.05) using a Shapiro-Wilk test. Data sets that met normality criteria were compared using a paired t-test, or two-sample t-test. Data sets that did not meet normality criteria were compared using a paired sample Wilcoxon test or Mann-Whitney test for unpaired samples. Data are reported as mean ± standard deviation (unless otherwise indicated). For box plots, the means are indicated by the filled square, medians are indicated by the horizontal bar, and 5–95% ranges are indicated by whiskers.

Figures were created using Origin 8.1 and PowerPoint (Microsoft, Redmond, WA).

### Reagents

All chemical reagents used to make the brain slicing and artificial cerebrospinal fluid (ACSF) solutions were obtained from Sigma-Aldrich (St. Louis, MO).

## Data Availability

All data generated or analysed during this study are included in the manuscript.

## Additional files

MDAR checklist
