# Supplementary figures and images for: The Na+/K+ pump dominates control of glycolysis in hippocampal dentate granule cells
Source: eLife. 2022 Oct 12;11:e81645. doi: 10.7554/eLife.81645 (PMC9592084; doi:10.7554/eLife.81645)

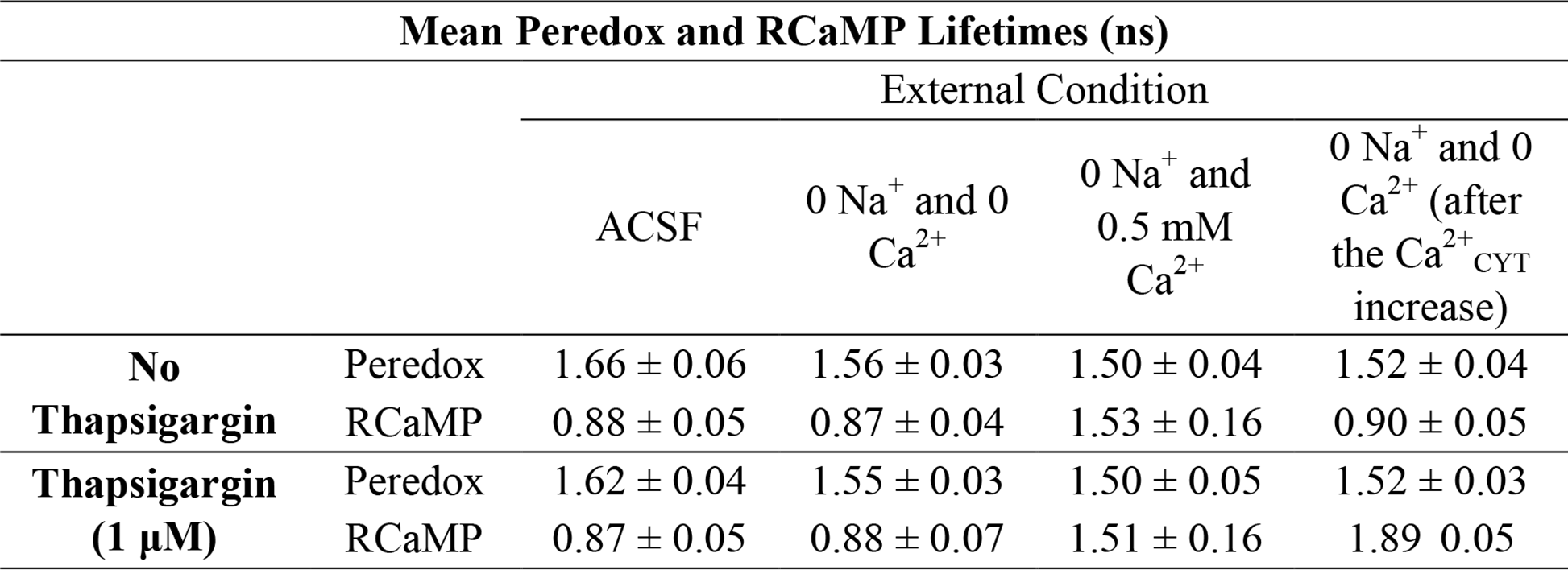

Supplement: Figure 1—figure supplement 1—source data 1. [file elife-81645-fig1-figsupp1-data1.zip › Figure 1 Supplement 1 Source Data 1.tif]

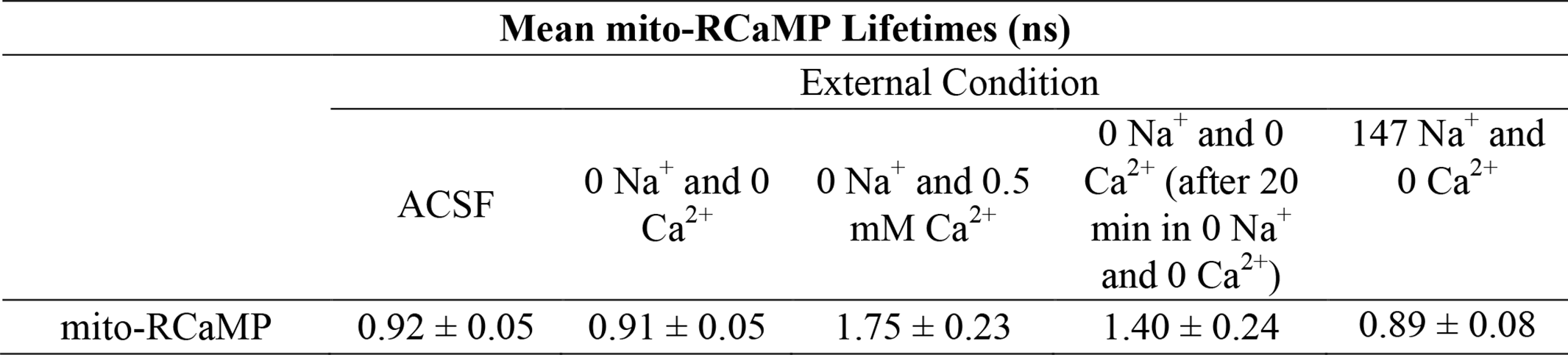

Supplement: Figure 1—figure supplement 3—source data 1. [file elife-81645-fig1-figsupp3-data1.zip › Figure 1 Supplement 3 Source Data 1.tif]
